# Structural basis for the toxin-coregulated pilus–dependent secretion of *Vibrio cholerae* colonization factor

**DOI:** 10.1126/sciadv.abo3013

**Published:** 2022-10-14

**Authors:** Hiroya Oki, Kazuki Kawahara, Minato Iimori, Yuka Imoto, Haruka Nishiumi, Takahiro Maruno, Susumu Uchiyama, Yuki Muroga, Akihiro Yoshida, Takuya Yoshida, Tadayasu Ohkubo, Shigeaki Matsuda, Tetsuya Iida, Shota Nakamura

**Affiliations:** ^1^ Department of Infection Metagenomics, Genome Information Research Center, Research Institute for Microbial Diseases, Osaka University, Osaka, Japan.; ^2^ Graduate School of Pharmaceutical Sciences, Osaka University, Osaka, Japan.; ^3^ Center for Infectious Disease Education and Research, Osaka University, Osaka, Japan.; ^4^ Graduate School of Engineering, Osaka University, Osaka, Japan.; ^5^ Department of Creative Research, Exploratory Research Center on Life and Living Systems (ExCELLS), National Institutes of Natural Sciences, Aichi, Japan.; ^6^ U-Medico Inc., Suita, Osaka, Japan.; ^7^ Department of Bacterial Infections, Research Institute for Microbial Diseases, Osaka University, Osaka, Japan.; ^8^ Integrated Frontier Research for Medical Science Division, Institute for Open and Transdisciplinary Research Initiatives, Osaka University, Osaka, Japan.

## Abstract

Colonization of the host intestine is the most important step in *Vibrio cholerae* infection. The toxin-coregulated pilus (TCP), an operon-encoded type IVb pilus (T4bP), plays a crucial role in this process, which requires an additional secreted protein, TcpF, encoded on the same TCP operon; however, its mechanisms of secretion and function remain elusive. Here, we demonstrated that TcpF interacts with the minor pilin, TcpB, of TCP and elucidated the crystal structures of TcpB alone and in complex with TcpF. The structural analyses reveal how TCP recognizes TcpF and its secretory mechanism via TcpB-dependent pilus elongation and retraction. Upon binding to TCP, TcpF forms a flower-shaped homotrimer with its flexible N terminus hooked onto the trimeric interface of TcpB. Thus, the interaction between the minor pilin and the N terminus of the secreted protein, namely, the T4bP secretion signal, is key for *V. cholerae* colonization and is a new potential therapeutic target.

## Teaser

The interaction of TCP and the N terminus of colonization factor is crucial for the efficient colonization of *V. cholerae*.

## INTRODUCTION

*Vibrio cholerae* is a comma-shaped gram-negative bacterium that causes cholera, a severe acute diarrheal illness (*1*, *2*). The *V. cholerae* O1 classical biotype led to six previous pandemics, whereas the O1 El Tor biotype is responsible for the seventh ongoing cholera pandemic, which began in 1961 (*3*–*5*). These strains carry the genes encoding virulence factors, including cholera toxin (CT), which is transported from the periplasm to the extracellular space via the type II secretion system (T2SS) (*6*–*10*), and toxin-coregulated pilus (TCP) (*11*–*15*). The T2SS is a supermolecular complex that assembles the pseudopilus, which consists of multiple pseudopilins. The pseudopilus is considered to act as a piston to extrude the CT through the secretin channel (*16*).

TCP is a member of the type IV pilus (T4P) family, which is evolutionarily, structurally, and functionally related to T2SS (*17*) and is important for promoting the uptake of the lysogenic bacteriophage CTXφ encoding CT. This pilus family is further divided into three subfamilies: T4aP, T4bP, and T4cP; TCP is categorized as T4bP (*17*). Similar to the T2SS, the T4P system has the ability to retract the pilus filament. For this purpose, the T4aP systems have a retraction adenosine triphosphatase (ATPase) but T4bP systems do not; however, recent experimental evidence shows that T4bP is capable of pilus retraction (*12*).

**Fig. 1.**
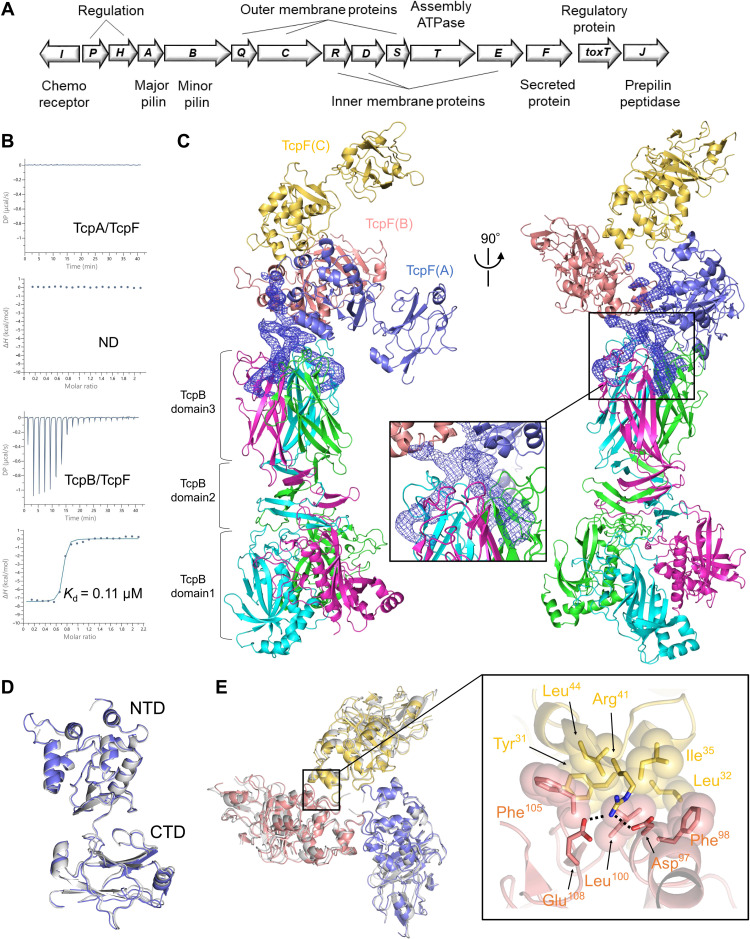
Trimeric structure of TcpF bound to the TcpB homotrimer. (**A**) Genetic organization of the *tcp* operon. Predicted functions of each gene product are indicated at the top and bottom of the figure. (**B**) The isothermal titration calorimetry (ITC) profiles depict TcpF titration with TcpA (top) or with TcpB (bottom). The lower panels present an integrated heat plot for the titration. (**C**) Crystal structure of the TcpB homotrimer in complex with three TcpF molecules. Front view (left) and side view (right) of the structure with the TcpB trimer (cyan, magenta, and green) and bound TcpF trimer (blue, salmon pink, and yellow) are shown as a ribbon representation. Close-up view of the residual electron densities (blue, countered at 1.0 σ) corresponding to the N-terminal portion of TcpF interacts with the clefts at the interfaces of the TcpB trimer (center). (**D**) Superposition of one TcpF molecule [TcpF(A), blue] in the TcpB-TcpF complex on TcpF crystal structure [gray, Protein Data Bank (PDB) code: 3oc5] shown by the ribbon model. (**E**) Superposition of TcpF trimer (blue, yellow, and salmon pink) in the TcpB-TcpF complex on the symmetrical TcpF trimer (gray) by interacting with symmetry-related molecules (left). Close-up view of hydrophobic and electrostatic interactions in the TcpF trimer (right). The residues involved in the interactions are presented as stick models, and the hydrophobic residues are presented as overlaid sphere models.

Similar to other T4bP members that are mostly produced by enteric pathogens (*15*, *17*), TCP plays an essential role in *V. cholerae* colonization and pathogenicity (*11*, *18*–*21*); 15 genes arranged into the *tcp* operon are putatively involved in its biosynthesis (Fig. 1A) (*11*, *15*). The two structural TCP components, the major pilin TcpA and minor pilin TcpB, are encoded on the *tcp* operon (*11*). Repetitive TcpA units form the filament structure, whereas TcpB displays multiple functions, such as efficient pilus assembly (*22*), pilus retraction (*22*), and CTXφ uptake (*23*). TcpB comprises three domains, of which the crystal structures of C-terminal domains (domain 2 and domain 3) were recently resolved, showing that these two domains consist of β-strand–rich folds forming a homotrimeric pilus initiation complex (*23*). This complex shows a marked similarity to that of the minor pilin CofB of another known T4bP: colonization factor antigen III (CFA/III) of enterotoxigenic *Escherichia coli* (ETEC) (*23*, *24*). The structure of TcpB domain 1 remains unknown and is predicted to be a pilin-like domain based on the similarity of its sequence with other T4b pilins. Domain 1 of TcpB presumably interacts with major pilin TcpA at the pilus tip and is also incorporated into the growing pilus of TCP, which may regulate pilus elongation and retraction by blocking the passage of pilus subunits through the secretin channel (*18*).

TCP elongation and retraction are important not only for CTXφ uptake but also for the secretion of a soluble colonization factor, TcpF, encoded on the *tcp* operon (*25*). Although its N-terminal region of TcpF may be required for its extracellular secretion, and Tyr^5^ in the corresponding region is the major secretion determinant (*26*), the underlying molecular mechanisms remain elusive. Furthermore, a TcpF deletion mutant facilitates normal TCP formation and autoagglutination but is incapable of efficient colonization in the infant mouse cholera model (*25*). The crystal structure of TcpF shows that it forms an elongated bilobed structure with two domains, a globular N-terminal domain (NTD) and an immunoglobulin-like folded C-terminal domain (CTD) but without structural homology to any other known proteins (*27*). Despite its critical contribution to *V. cholerae* pathogenesis (*28*, *29*), the mode of TcpF secretion and induction of *V. cholerae* colonization remain unclear.

In the present study, by integrating the results from x-ray crystallography, physicochemical analyses, and structural modeling, we demonstrate that TcpF binds to TcpB at the tip of the TCP filament and present a structural model of this complex. This model allows us to propose a novel secretion mechanism for TcpF and provides structural insights into its role in *V. cholerae* colonization. We also define the T4bP secretion signal, a key binding motif for the interaction with minor pilin during T4bP-dependent secretion of a soluble colonization factor protein.

## RESULTS

### TcpF selectively binds to minor pilin TcpB of TCP

An immunoprecipitation assay using the cross-linked lysates of *V. cholerae* indicated that TcpF binds to TCP via its interaction with the major pilin TcpA (*26*). However, owing to the lower expression of the minor pilin TcpB, the possibility of interaction between TcpF and TcpB needed to be considered. Thus, we titrated TcpF with TcpA or TcpB using isothermal titration calorimetry (ITC). Unlike the previously reported results, TcpF was found to bind to the minor pilin TcpB with a dissociation constant (*K*_d_) of 0.11 μM, whereas no heat-induced change was observed in the binding of major pilin TcpA (Fig. 1B).

To clarify the interaction between TcpB and TcpF, we performed cocrystallization experiments with the TcpB-TcpF complex. The initial phases were solved to a resolution of 4.05 Å by molecular replacement (MR) using the reported TcpF structure and TcpB structure determined here (details below) as search models (table S1) (*27*). The three TcpB molecules formed a trimer in an asymmetric unit. In addition, three TcpF molecules were found above the C-terminal domain 3 of TcpB (Fig. 1C). This feature differed from that of the ETEC CofB-CofJ complex, in which minor pilin CofB interacts with the secreted protein CofJ at a 3:1 molar ratio (*30*). Every TcpF molecule was a bilobed structure constituting an NTD and a CTD connected by a short linker, with little interaction between them (Fig. 1D). These CTDs were spatially separated without direct contact. The structural analysis suggested the existence of a TcpF trimer shaped like a flower with three petals and an N-terminal flexible extension, which partly interacts with the clefts at the interfaces of the TcpB trimer (Fig. 1C). However, the model building of the N-terminal binding region was unsuccessful due to the relatively low resolution of the present x-ray data (Fig. 1C).

The crystal structure of TcpF alone was previously described as a monomer in an asymmetric unit (*27*). However, TcpF interacts with symmetry-related molecules to form a symmetrical trimer with a conformation nearly identical to that observed in the TcpF-TcpB structure (Fig. 1E). The TcpF trimer formed via an interaction with the NTD, where the interaction surfaces primarily consisted of hydrophobic and aromatic residues, such as Tyr^31^, Leu^32^, Ile^35^, Leu^44^, Phe^98^, Leu^100^, and Phe^105^, forming a hydrophobic core accompanied by some polar residues making a network of salt bridges between Arg^41^, Asp^97^, and Glu^108^ (Fig. 1E).

### Crystal structures of TcpB in complex with a TcpF (1–15) peptide

To investigate the mechanism underlying the interaction between TcpB and the N terminus of TcpF, we synthesized a TcpF (1–33) peptide comprising the N-terminal 33 residues before Cys^34^ that forms a disulfide bond with Cys^47^. This peptide bound to TcpB with an affinity of *K*_d_ = 5.2 μM (fig. S1). The absence of globular domains of TcpF resulted in a 50-fold decrease in affinity, signifying the additive role played by the NTD. We then synthesized the N- or C-terminal truncated peptides to elucidate the core fragment of TcpF (1–33) responsible for the interaction. The C-truncated peptides, TcpF (1–20) and TcpF (1–15), bound to TcpB with binding affinities comparable to the TcpF (1–33) peptide, while the affinity of the TcpF (1–10) peptide drastically decreased (fig. S1). Our previous study has shown that the substitution of Phe^10^, corresponding to Tyr^5^ in TcpF, to Ala in the ETEC CofJ peptide abolished its binding affinity with the minor pilin CofB (*30*). Hence, we prepared a Y5A variant of the TcpF N-terminal fragment (1–15) and confirmed its inability to bind TcpB, indicating that TcpF-Tyr^5^ plays a key role in binding with TcpB (fig. S1). Accordingly, the N-truncated peptide, TcpF (6–33), completely lost its binding affinity for TcpB (fig. S1).

**Fig. 2.**
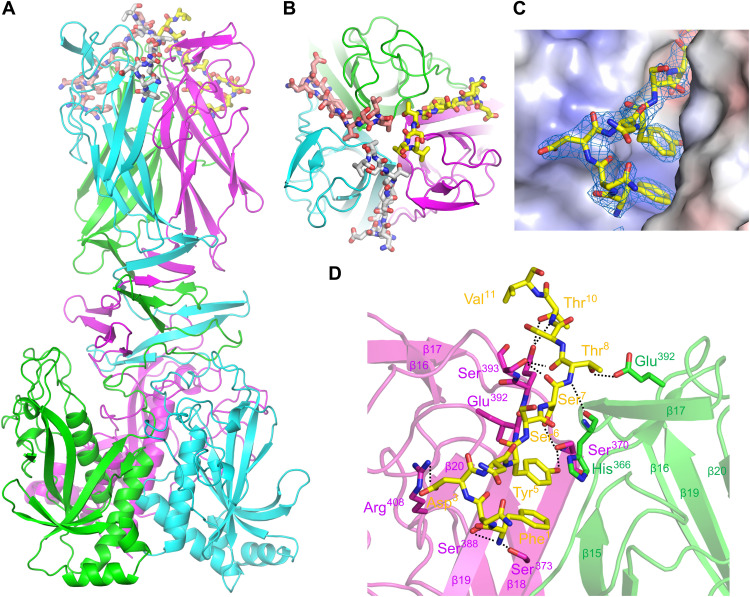
Crystal structure of the TcpB-TcpF (1–15) complex. (**A**) Side view of the structure with the TcpB trimer (cyan, magenta, and green) depicted as ribbons, and bound TcpF (1–15) peptides (white, yellow, and salmon pink) represented by bold stick models. (**B**) Top view of the TcpB-TcpF (1–15) complex structure. (**C**) Electrostatic surface potential of the TcpF (1–15) binding site. TcpF (1–15) peptides can be recognized by clefts formed by TcpB domain 3, forming a unique hook-like conformation. 2 m*F*o-D*F*c omit map contoured at 2.0 σ corresponding to peptide-binding grooves. (**D**) Interactions between two TcpB molecules (green and magenta) and one TcpF (1–15) peptide (yellow). Residues involved in the interactions are depicted as stick models.

We then analyzed the structure of TcpB alone and in complex with the TcpF (1–15) peptide. The apo-TcpB structure was determined by using selenomethionine derivatives before further refinement to a resolution of 2.32 Å using data obtained from the crystal of the wild-type protein (fig. S2 and table S1). We used this TcpB structure as an MR search model to determine the TcpB and TcpF (1–15) peptide complex at a resolution of 2.30 Å (table S1 and Fig. 2, A to C). Two nearly identical copies of the TcpB trimer were located, with a C-alpha root mean square deviation of 0.642 (fig. S3); the remaining electron densities, corresponding to six TcpF (1–15) peptides, were observed on each cleft sandwiched by two molecules of domain 3 in the TcpB trimer. A structural model for all six peptides was successfully built, except for residues 12 to 15 on the C terminus due to the disorder of the region (Fig. 2D). Superimposing the six TcpF (1–15) peptides showed the equivalence of peptide-binding modes at each cleft (fig. S3).

A unique hook-like conformation, in which the five N-terminal residues of TcpF adopt a type I β turn structure stabilized by pi-pi stacking of the Phe^1^-Tyr^5^ pair (Fig. 2, B to D), was recognized by the TcpB trimer. In forming this rather compact turn structure, the bulky aromatic amino acid pair got embedded in the binding pocket at the bottom of the clefts, with its N terminus firmly docked at β18 and β19 of TcpB via hydrogen bond interactions between the Phe^1^ amine group (TcpF) and the side chains of Ser^373^ and Ser^388^ (TcpB) (Fig. 2D). The electrostatic interaction between Asp^3^ (TcpF) and Arg^408^ (TcpB), as well as the stacking interaction between Tyr^5^ (TcpF) and His^366^ (TcpB), further strengthened the rigidity of the turn structure (Fig. 2D). Notably, the residues from Ser^6^ to Val^11^ at the upper half of the cleft formed an extended conformation that fitted well to the slope-like cleft, with an extensive network of hydrogen bonds (Fig. 2D). We speculate that the C terminus of TcpF (1–15) may help their NTDs efficiently come into contact to form a symmetrical trimer (Fig. 1, C and E). Using the structural model of the TcpB-TcpF (1–15) complex, we re-refined the whole TcpB-TcpF hexameric complex structure determined at a resolution of 4.05 Å (fig. S4).

### TcpB-TcpF complex association in solution

**Fig. 3.**
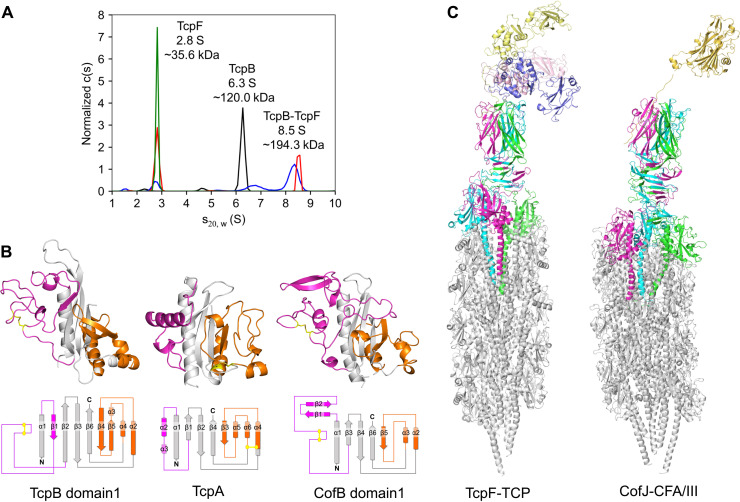
T4bP structural models. (**A**) Analytical ultracentrifugation of TcpF alone, TcpB alone, and mixtures of TcpB and TcpF. The c(s) distribution profile based on sedimentation velocity data collected at 42,000 rpm and 20°C is shown. The spectra of 20 μM TcpF alone, 5 μM TcpB alone, a mixture of 5 μM TcpB/5 μM TcpF, and a mixture of 5 μM TcpB/20 μM TcpF are shown in green, black, blue, and red, respectively. TcpF and TcpB exist as single species with a sedimentation coefficient of 2.8 and 6.3 S, respectively. (**B**) Structural comparison between TcpB domain 1 and T4bP pilin domains in TCP or CFA/III. Crystal structures of previously determined major pilin subunit, TcpA (PDB code: 1oqv), and minor pilin subunit, CofB domain 1 (PDB code: 5ax6); structural model (above) and topology diagram (below). The D-region and α/β loop are represented in orange and purple, respectively. The hydrophobic structural core is depicted in gray. Disulfide bonds are represented by a yellow stick model. In general, type IV pilins form a disulfide bond in the D-region; however, TcpB has a disulfide bond between Cys^85^ and Cys^107^ in the α/β loop instead of the D-region. (**C**) Left: Side view of TcpF-TCP depicted as a ribbon model, built by superimposing the crystal structure of TcpB-TcpF onto the TCP model. Right: Side view of the CofJ-CFA/III pilus depicted as a ribbon model, built by superimposing the crystal structure of CofJ (*1*–*24*)–CofB onto our previously reported CFA/III pilus model.

To confirm the association of TcpB and TcpF in solution, we performed sedimentation velocity-analytical ultracentrifugation. This experiment demonstrated that TcpB (43.5 kDa) is a trimer with an estimated molecular mass of 120.0 kDa (Fig. 3A). On the other hand, TcpF (35.8 kDa) is a monomer with an estimated molecular mass of 35.6 kDa (Fig. 3A). When mixing TcpB and TcpF at an equimolar concentration of 5 μM, we observed three species: TcpB, TcpF, and the TcpB-TcpF complex (Fig. 3A). In case of an excess (20 μM) of TcpF mixing with TcpB, almost all TcpB formed a complex with an apparent molecular weight of 194.3 kDa, close to the theoretical value (202.1 kDa) for a TcpB-TcpF complex at a 3:2 stoichiometry (Fig. 3A). This value contradicts the observation of a 3:3 complex (237.9 kDa) in the TcpB-TcpF crystal. The smaller apparent molecular weight than the actual molecular weight of the complex could be observed for the interaction with a fast dissociation rate constant (*31*). Therefore, we performed native mass spectrometry on the TcpB-TcpF complex and found that almost all TcpB-TcpF complexes were formed with a 3:3 stoichiometry (fig. S5). These results indicate that TcpF exists as a monomer in solution; its trimerization is promoted only in the presence of a TcpB trimer.

The trimerization of TcpF is stabilized by the hydrophobic core of the NTD (Fig. 1E). Of the residues comprising the core of the NTD, Leu^100^ is completely conserved in the pathogenic strains of *V. cholerae* (fig. S6) (*26*). To evaluate the contribution of this core to the trimerization, we prepared a TcpF-L100D mutant. The mass spectrum of the mutant showed that the peaks of the hexamer complex decreased drastically compared with those in the spectrum of the wild type; by contrast, those of the TcpB trimer alone increased (fig. S7). In the spectrum of the mutant, we observed the minor peaks with the following TcpB-TcpF stoichiometries: 3:1, 3:2, and 3:3. The ITC experiment showed that the TcpF-L100D was bound to TcpB with an affinity of *K*_d_ = 1.9 μM, comparable to that of the TcpF (1–33) peptide (fig. S8). We confirmed that a TcpF-Y5A mutant lacking the N-terminal interaction lost the binding affinity for TcpB (fig. S8), and no hexametric complex was observed in the mass spectrum of TcpB-TcpF-Y5A (fig. S7). These results suggest that the N-terminal interaction promotes the associations of three TcpF molecules and that the additional NTD-dependent associations are important for the stable formation of the flower-shaped hexametric TcpB-TcpF complex.

### Structural model of the TcpF-TCP filament complex

To build the whole structural model of the TcpF-TCP complex, we performed computational modeling of a major pilin TcpA filament by fitting the previously reported crystal structure of TcpA to TCP electron microscopy density map reported by Craig and colleagues (*32*) (fig. S9). We then replaced three helically arranged TcpA molecules at the tip by three TcpB pilin-like domains (domain 1) (fig. S9). The structure of TcpB domain 1 adopts an α/β-roll fold similar to that of other type IV pilins (*24*, *33*, *34*), but the α/β loop connecting the α1 helix to the central β-sheet is outstandingly different (Fig. 3B). The α/β loop of TcpB consists of 64 residues, which are remarkably longer than the 38 residues of major pilin TcpA, and has a disulfide bond formed between Cys^85^ and Cys^107^ in the loop (Fig. 3B). This disulfide bond in the α/β loop can also be observed in domain 1 of ETEC minor pilin CofB and likely stabilizes the longer α/β loops of these minor pilins (Fig. 3B).

TcpB domain 1 interacts with TcpA at the pilus tip in a manner similar to that of TcpA in a TcpA filament (fig. S9). In addition to the interaction between the positively charged amine of the N terminus and Glu^5^ of the two α1s, the TcpB-TcpA interaction is presumably promoted by the shape and charge complementarity of globular domains (see fig. S9 and the captions for detail) (*32*, *35*). Since TcpA filament is composed of a three-stranded helical arrangement with an axial increase of 8.4 Å (*32*), the three molecules of TcpB domain 1 are also arranged helically. The linker region between TcpB domains 1 and 2 (Thr^230^-Ala^242^) is composed of 13 residues, including two Gly and one Ser, and is both flexible and long enough to connect the furthest domain 1 (fig. S10). Last, the TcpF-TCP filament model was constructed by linking the remaining parts of the TcpB-TcpF hexameric crystal structure at the tip of the TCP filament model. The resulting model was energy minimized to obtain a stable structure (Fig. 3C). The unique trimeric association of TcpF at the distal pilus end largely contrasts with that of the CofJ-CFA/III filament model (Fig. 3C).

### Bacterial species–dependent interaction between T4bP minor pilin and the cognate secreted protein

**Fig. 4.**
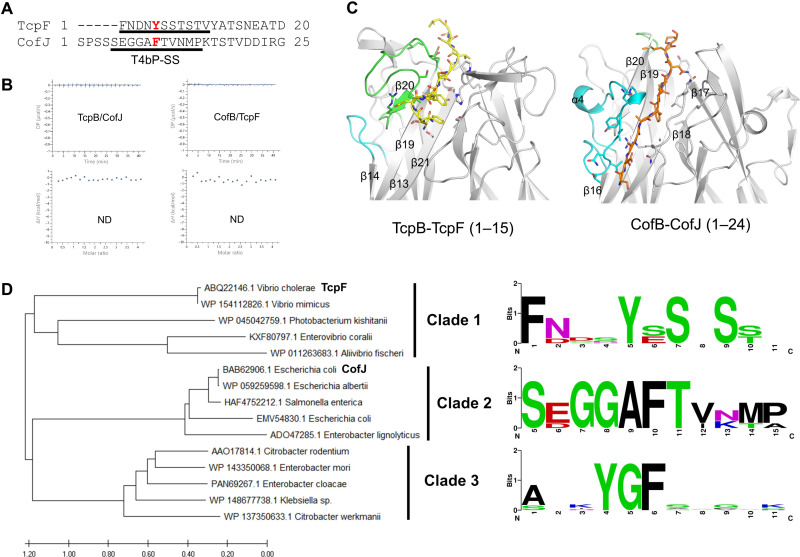
Minor pilin selectively binds to particular secreted proteins. (**A**) Protein sequence alignment of the N terminus based on T4bP-SS (underlined black) between ETEC CofJ and *V. cholerae* TcpF. Aromatic amino acids that are important for the interaction with each minor pilin are shown in red. Mature CofJ and TcpF N-terminal sequences generated from N-terminal signal sequence cleavage are predicted by SignalP 6.0 (https://services.healthtech.dtu.dk/service.php?SignalP). (**B**) ITC profiles of TcpF titration with CofB (right) or CofJ with TcpB (left). In each case, raw titration data (top) and integrated heat measurements for the titration (bottom) are presented. (**C**) Differences in the interactions between TcpB-TcpF (1–15) (left) and CofB-CofJ (1–24) (right). TcpB and CofB molecules are presented in gray, and TcpF (1–15) and CofJ (1–24) are presented as stick models in yellow and orange, respectively. The β19/β20 loop in TcpB and the corresponding loop in CofB (β19/β20) are shown in green. The α4/β16 loop in CofB and the corresponding loop in TcpB (β13/β14) are shown in cyan. The residues involved in the interactions are depicted as stick models. (**D**) Left: Phylogenetic tree of TcpF and other (predicted) secreted proteins. The protein sequences of the mature secreted proteins were analyzed and used to construct a phylogenetic tree showing the three main clades. Right: Sequence logo plot of (predicted) T4bP-SS in clade 1 (top), clade 2 (middle), and clade 3 (bottom). The sequences used in the analysis are shown in fig. S11. These figures were created by WebLogo (https://weblogo.berkeley.edu/logo.cgi).

Despite the structural differences between the globular domains of TcpF from *V. cholerae* and CofJ from ETEC (*36*), the N-terminal–dependent interaction with the minor pilin is conserved. Furthermore, given the conserved concave-like binding pocket in both minor pilin trimers, the recognition of aromatic residues by the trimer-dependent formation of the binding pocket may be a common feature of the T4bP system. We then aligned the minor pilin binding sequence of these secreted proteins, i.e., Phe^1^-Val^11^ for TcpF and Ser^5^-Pro^15^ for CofJ, to characterize the binding motif (hereafter, these peptide segments, each of which encompasses a critical aromatic residue, will be referred to as type IVb pilus secretion signal T4bP-SS). However, no obvious sequence similarity was found between them (Fig. 4A). To examine cross-reactivity, we performed ITC experiments, revealing no binding between TcpF and CofB or between CofJ and TcpB (Fig. 4B).

The structure of the CofB-CofJ complex demonstrated that, upon binding to the CofB trimer, the T4bP-SS of CofJ forms an elongated structure, sandwiched between two loops, α4/β16 and β17/β18, at the binding cleft (Fig. 4C). These loops are in close contact with CofJ T4bP-SS at the region spanning three consecutive residues, Gly^7^, Gly^8^, and Ala^9^, based on main-chain atom recognition (*30*). Thus, amino acids with bulky side chains at those positions likely preclude interactions at the cleft. Although TcpF T4bP-SS contains Tyr^5^, which could assume a position similar to that of CofJ Phe^10^, differences between the corresponding residues (Asn^2^, Asp^3^, and Asn^4^ in TcpF compared with Gly^7^, Gly^8^, and Ala^9^ in CofJ, respectively) hamper the interaction between TcpF and CofB (Fig. 4A). With respect to TcpB and CofJ, although both their domain 3 structures adopt a similar H-type lectin-like fold, the length of the β13/β14 loop of TcpB, corresponding to the α4/β16 loop of CofB, is outstandingly short. This difference in the surface-exposed loop in TcpB makes it incapable of accommodating the elongated structure of CofJ T4bP-SS, which may explain the lack of binding between TcpB and CofJ (Fig. 4C).

The minor pilins, TcpB and CofB from *V. cholerae* and ETEC, respectively, interact with the T4bP-SS of their cognate secreted proteins (Figs. 1B and 4B). The T4bP-SS of *V. cholerae* and close-related species has the motif “FxxxYxSxS,” and Phe^1^ and Tyr^5^ are conserved among members of the *Vibrionaceae* family, such as *Enterovibrio coralii*, *Aliivibrio fischeri*, *Vibrio mimicus*, and *Photobacterium kishitanii* (Fig. 4D and fig. S11), indicating that the T4bP-SS of the secreted protein of these bacteria could also form a similar turned structure and bind to the corresponding minor pilin. Phe^10^ in CofJ is also conserved among *Escherichia albertii*, *Enterobacter lignolyticus*, and *Salmonella enterica* in the Enterobacteriaceae family (Fig. 4D and fig. S11). The T4bP-SS of this family has the motif “SxGGAFT,” and the small side-chain amino acids (Ser, Gly, and Ala) in the first half of the motif might be optimized to fit into the narrow groove formed by the two long loops as those observed in CofB (α4/β16 loop and β17/β18 loop) (Fig. 4C). In another clade of Enterobacteriaceae, the motif “YGF” is found among *Citrobacter rodentium* (*37*), *Enterobacter mori*, *Enterobacter cloacae*, *Citrobacter werkmanii*, and *Klebsiella* sp. (Fig. 4D and fig. S11). The completely conserved aromatic amino acids Tyr^4^ and Phe^6^, or Tyr^5^ in TcpF and Phe^10^ in CofJ, are presumed to play an important role in the interaction with the cognate minor pilin. These results indicate that the T4bP of each bacteria family has evolved to recognize the T4bP-SS of its cognate secreted protein.

## DISCUSSION

Here, we revealed that the *V. cholerae* colonization factor TcpF interacts with minor pilin TcpB at a 3:3 molar ratio via its N-terminal T4bP-SS. The resulting TcpB-TcpF hexameric complex passes through the secretin ring in the outer membrane, helped along by TCP filament elongation. The three-dimensional structure of secretin TcpC multimers remains unsolved. However, the in situ structure of the TCP machine in its piliated state has been previously investigated using electron cryotomography (*13*). In the electron density map, the TCP filament was observed to fit the secretin ring, suggesting that the ring diameter is similar to that of the TCP filament, which is approximately 80 to 90 Å, as inferred from our current filament model (fig. S12). In contrast, the TcpF trimer observed in the crystal shows a maximum diameter of approximately 110 Å and is apparently larger than the secretin ring (fig. S12). This requires certain conformational changes in either secretin, TcpF, or both. Since the TcpF-NTD and CTD are connected by a short linker, at least in TcpF, this structural flexibility would allow these two domains to temporarily change their relative configuration, reminiscent of petals closing on a flower, making it small enough to pass through the secretin ring.

At the extracellular milieu, this flower-shaped TcpF trimer is of functional importance in *V. cholerae* colonization. Megli and Taylor (*26*) previously reported that *V. cholerae* strains expressing derivatives of the NTD, such as TcpF:ΔAA28–31 (deletion from 28 to 31) and TcpF:ΔAA99–100 (deletion from 99 to 100), cannot colonize an infant mouse model of *V. cholerae*. Furthermore, our structural analysis demonstrated that these two deleted regions, which are conserved strictly among pathogenic *V. cholerae* strains (fig. S6) (*26*), face each other at the trimerization interface to form the hydrophobic core (Fig. 1E). Although it still has binding potency via its T4bP-SS, the substitution of Leu^100^ to alanine in TcpF disrupts its trimeric associations of NTDs in the presence of TcpB (figs. S7 and S8). Therefore, the trimerization of TcpF via its NTDs might be essential for its function in colonization.

Since TcpF exists as a monomer in solution, the trimerization via its NTD is promoted only in the presence of TcpB and enhances the efficiency of TcpF secretion, allowing three molecules at a time. Nevertheless, under the conditions immediately following the secretion, the submicromolar affinity of TcpF toward TcpB is unable to maintain the hexameric TcpB-TcpF complex stably and dissociates them readily from TcpB (figs. S7 and S8). Therefore, to maintain TcpB in its fully complex state, an excess amount of TcpF should be present in the immediate vicinity of the TcpB trimer. It has been experimentally demonstrated that a large amount of TcpF is secreted out of the bacteria, comparable to that of the CT secreted by the T2SS apparatus (*25*). A TcpF secretion model that requires repeated cycles of TCP elongation and retraction, similar to the piston model in T2SS, has been proposed (*26*). Since no retraction ATPase has been found in both the T2SS and T4bP systems, the mechanisms of retraction have not been conclusively determined. On the basis of a micropillar assay, one study has recently established the existence of the retraction process and showed that the TcpB-E5V mutant, which is unable to interact with the Met^1^ amine of TcpA, forms TCP, but the efficiency of TCP retraction and TcpF secretion are greatly reduced (*12*). The researchers proposed an ATPase-independent retraction model in which TcpB is incorporated into the bottom of the elongated TCP filament, and then the assembly is blocked via unknown mechanisms. The crystal structure of TcpB domain 1 that was determined here allows us to simulate this situation by replacing one TcpA molecule at the bottom of the TCP filament model with one TcpB subunit. In this model, Glu^5^ of TcpB can interact with the Met^1^ amine of TcpA. However, the α/β loop of TcpB collides with the D-region of TcpA (fig. S13). A drastic structural change in the loop is required for binding to TcpA without steric hindrance; however, this is unlikely because the disulfide bond (Cys^85^-Cys^107^) stabilizes the α/β loop structure. Notably, the C85A or C107A mutant of TcpB significantly impairs TCP functions expected to be involved in retraction, such as TcpF secretion, autoagglutination, and transduction efficiency (*38*). These findings suggest that TCP retraction is triggered by steric hindrance between domain 1 of TcpB and TcpA.

**Fig. 5.**
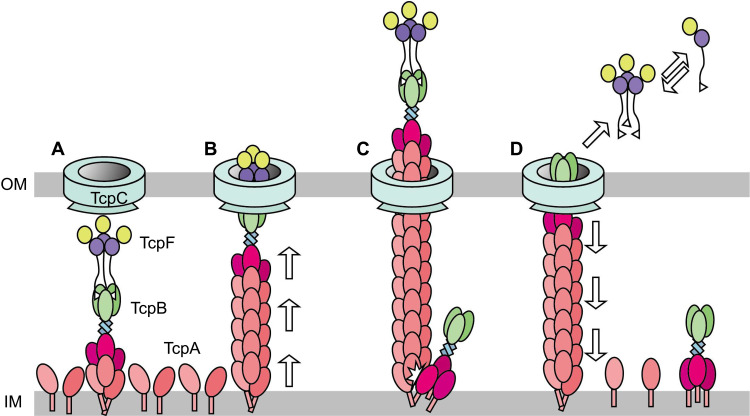
Model of the T4bP system transporting TcpF. (**A**) TcpF forms a trimer, and its flexible N-terminal T4bP-SS interacts with the TcpB trimer located on top of the pilus filament. (**B**) The pilus filament elongates by the addition of TcpA molecules and transports TcpF from the periplasm through the secretin ring TcpC out of the bacteria. (**C**) Adding domain 1 of TcpB to the bottom of the TCP filament causes steric hindrance, initiating pilus retraction. (**D**) The TcpF trimer is detached from TcpB and dissociates to monomers.

On the basis of these findings, we proposed a model for TcpF secretion by TCP retraction. Once TcpF binds to TcpB (Fig. 5A), the assembling of TcpA elongates the pilus, which simultaneously carries TcpF and reaches the secretin ring (Fig. 5B). During the translocation of secretin, the two domains of TcpF may temporarily change their relative arrangements, allowing the resulting compact structure to pass through the ring (Fig. 5B). Then, TcpF dissociates from the TcpB trimer and is deposited around the bacterium (Fig. 5, C and D). The incorporation of domain 1 of TcpB into the lowest part of the elongated TCP filament induces steric hindrance and initiates the retraction of the pilus (Fig. 5C). TcpA molecules dissociate by retraction, dissolve back into the inner membrane, and are repeatedly reused for TCP assembly while secreting TcpF, thereby accumulating TcpF in the proximity of the bacterium (Fig. 5D). As evidenced by the analytical ultracentrifugation experiments (Fig. 3A), the increased concentration of TcpF saturates the three equivalent binding sites of the TcpB trimer to form the heterohexamer complex that can recognize the target membrane(s) of host and/or *V. cholerae* cells. We speculate that this concentration-dependent formation of a productive complex also acts as a functional switch that plays an important role in sensing highly confined spaces, such as intestinal crypts (*39*), for efficient *V. cholerae* colonization against the flow generated by peristalsis. Although the target molecule(s) of TcpF remains unclear, it has been reported that the adhesion of *V. cholerae* to the infant mouse cholera model requires Glu^251^, Glu^252^, and Tyr^292^ of the CTD (*26*, *27*). Our structural analyses revealed that these residues are located on the inner side of the three-petaled flower structure of TcpF (fig. S14), suggesting that it may recognize the trimeric receptor(s) by those residues located at the inner side of the trimeric structure.

In the post-antibiotic era, there is an increasing interest in anti-adhesive agents targeting bacterial adhesins and colonization factors (*40*–*42*). The interaction between the minor pilin and the secreted protein T4bP-SS identified here may be an attractive target for developing anti-adhesive agents against *V. cholerae* to inhibit TcpF secretion and adhesion processes. Each T4bP-SS diversified according to bacteria type during evolution; this diversity may be advantageous for bacterial infection and survival. Elucidating the structural basis of the interaction between various T4bP-SSs and minor pilins may provide unique design principles for novel anti-adhesion agents that selectively inhibit or modulate infection of T4bP-producing bacteria.

## MATERIALS AND METHODS

### Cloning, expression, and purification of recombinant proteins

We designed N-terminal TcpA and TcpB truncated constructs, from which the first 28 residues were excluded to solubilize the recombinant protein, and a TcpF construct with a deletion of the Sec signal sequence from Met (−20) to Ala (−1). The sequences encoding TcpA, TcpB, and TcpF were polymerase chain reaction (PCR)–amplified from the genomic DNA of the classical *V. cholerae* O1 strain 569 B of (RIMD 2203107) using the listed primer pairs (table S2). The PCR products were digested with Msc I and Xho I (New England Biolabs) and ligated into the modified pET-44 expression vector, resulting in a thioredoxin tag, a 6× His tag, and a tobacco etch virus (TEV) protease recognition sequence at the N terminus. After purifying the resulting TcpF, the vector-derived sequence Gly-Gly remained at the N terminus. To exclude this extra sequence, a TcpF sequence with a factor Xa protease recognition sequence added to the N terminus was amplified from the Gly-Gly-TcpF (GG-TcpF) expression plasmid via inverse PCR using a TcpFF_forward primer and a phosphorylated TcpFF_reverse primer (table S2). Amplified linear DNA-encoded TcpF was self-ligated. *E. coli* SHuffle T7 Express LysY cells (New England Biolabs) were transformed with the resulting expression vectors and grown in Luria-Bertani medium containing ampicillin (100 μg/ml). Cells collected from the induced culture were lysed via sonication, and then the supernatant was applied to a HisTrap FF crude column. The thioredoxin tag was cleaved using TEV protease for TcpA, TcpB, and factor Xa protease for TcpF. Thereafter, the protein was further purified using a HiTrap Q HP anion-exchange column and a HiLoad 16/600 Superdex 200 pg size exclusion column (GE Healthcare Biosciences) pre-equilibrated with a buffer containing 20 mM tris-HCl and 150 mM NaCl (pH 8.0). The SeMet derivative of TcpB was prepared according to a previously reported method using SeMet minimal medium (*43*). The purification procedure of the SeMet-derivatized protein was similar to that used for TcpB. For purification of the TcpF-TcpB complex, TcpF was mixed with TcpB and subsequently allowed to rest for 1 hour on ice and then applied onto a HiLoad 16/600 Superdex 200 pg size exclusion column pre-equilibrated with buffer as described above. Purified proteins were analyzed via SDS–polyacrylamide gel electrophoresis.

The plasmids for the expression of the TcpF-Y5A and TcpF-L100D mutants were prepared using the TcpF-Y5A forward and reverse primers for the TcpF-Y5A mutant and the TcpF-L100D forward and reverse primers for the TcpF-L100D mutant using the quick-change method. Expression and purification of the mutants were performed as described for the wild-type TcpF.

### Isothermal titration calorimetry

All recombinant proteins and synthesized peptides were dissolved in a buffer containing 20 mM tris-HCl and 150 mM NaCl (pH 8.0). ITC experiments were performed using iTC200 (GE Healthcare Biosciences) or MicroCal PEAQ-ITC (Mulvern). In experiments using TcpF/TcpA, TcpF/TcpB, and TcpF-Y5A/TcpB, TcpF or TcpF-Y5A solution (80 μM) was loaded into the cell, and the TcpA or TcpB solution was loaded into the syringe (800 μM as a monomer). In experiments using TcpF-L100D/TcpB, the TcpF-L100D solution (63 μM) was loaded into the cell, and the TcpB solution was loaded into the syringe (800 μM as a monomer). In experiments using TcpF (1–33) peptide/TcpB, the TcpF (1–33) solution (50 μM) was loaded into the cell, and the TcpB solution was loaded into the syringe (768 μM as a monomer). In experiments using TcpB with the synthesized peptides, TcpF (6–33), TcpF (1–20), TcpF (1–10), and TcpF(1–15)-Y5A, the peptide solution (80 μM) was loaded into the cell and the TcpB solution was loaded into the syringe (800 μM as a monomer). In experiments TcpF (1–15)/TcpB, the TcpF (1–15) solution (20 μM) was loaded into the cell, while the TcpB solution was loaded into the syringe (800 μM) as a monomer. In experiments using TcpF/CofB and CofJ/TcpB, the TcpF or CofB solution (9.7 μM) was loaded into the cell, and the CofB or TcpB solution was loaded into the syringe (197 μM) as a monomer. All titrations were performed by sequentially titrating 1 μl for the first titration point and 2 μl for all subsequent titration points with the syringe solution at 120-s intervals at 25°C. The thermograms were analyzed with the MicroCal PEAQ-ITC Analysis Software (version 1.40).

### Analytical ultracentrifugation

Sedimentation velocity experiments were performed using an Optima analytical ultracentrifuge (Beckman Coulter) equipped with an eight-hole An-50 Ti rotor at 20°C using 3-mm or 12-mm double-sector charcoal-filled epon centerpieces with sapphire windows. The TcpB, TcpF, and TcpB-TcpF mixture was dissolved in phosphate-buffered saline at pH 7.4. Sedimentation data were collected at 42,000 rpm with a radial increment of 0.001 cm using absorbance optics. The detection wavelengths for 5 μM TcpB, 20 μM TcpF, the 5 μM TcpB/5 μM TcpF mixture, and the 5 μM TcpB/20 μM TcpF mixture were 234, 287, 238, and 289 nm, respectively. The 12-mm centerpieces were used for the measurements. The distribution of sedimentation coefficients was analyzed using the c(s) method in the SEDFIT program (*44*). The range of sedimentation coefficients for fitting was 0 to 15 S, with a 150 resolution. The buffer density and viscosity calculated using the SEDNTERP program (*45*) were 1.00564 g/ml and 1.0199 cP, respectively.

### Native mass spectrometry

The buffers for TcpF, the TcpF-Y5A mutant, the TcpF-L100D mutant, and TcpB were exchanged into 200 mM ammonium acetate (pH 7; Sigma-Aldrich) by passing the proteins through a Bio-Spin 6 column (Bio-Rad, 10-kDa cutoff). Buffer-exchanged TcpF or its mutants were mixed with TcpB and incubated at room temperature for 20 min to obtain mixtures of 20 μM TcpF/5 μM TcpB, 20 μM TcpF-Y5A/5 μM TcpB, 50 μM TcpF-Y5A/5 μM TcpB, and 20 μM TcpF-L100D/5 μM TcpB. These mixtures, 20 μM TcpF and 20 μM TcpF mutants, were analyzed by nano-electrospray ionization mass spectrometry with gold glass capillaries made in-house (5-μl sample loaded per analysis). Spectra were recorded on Q Exactive UHMR Hybrid Quadrupole-Orbitrap Mass Spectrometer (Thermo Fisher Scientific) in positive ionization mode at 1.2-kV spray voltage with 21.0-V source DC offset, 40-V hollow cathode discharge (HCD) voltage, and 5.0 trapping gas setting. The spectra were calibrated using cesium iodide (4 mg/ml) and analyzed using BioPharma Finder software (Thermo Fisher Scientific).

### Crystallization, data collection, and structure determination

Purified TcpB was mixed with the TcpF (1–15) peptide at a molar ratio of 1:3 for cocrystallization. Crystallization of TcpB, the SeMet derivative of TcpB, TcpB-TcpF (*1*–*15*), and TcpB-TcpF was performed using the sitting-drop vapor-diffusion method at 293 K. Crystals of TcpB and the SeMet derivative were obtained from a crystallization drop containing 4.0-μl protein solution (50 mg/ml) and 4.0-μl reservoir solution consisting of 0.1 M tris-HCl and 1.8 M lithium sulfate monohydrate (pH 8.8) within 3 months. Thereafter, cocrystals of TcpB-TcpF (1–15) were obtained from a crystallization drop containing 2.0-μl protein solution (50 mg/ml) and 2.0-μl reservoir solution comprising 0.1 M Hepes, 0.28 M CaCl_2_, and 22% (v/v) PEG400 (polyethylene glycol, molecular weight 400) (pH 7.5), within 1 week. TcpB-TcpF crystals were obtained from a crystallization drop containing 1.0-μl protein solution (50 mg/ml) and 1.0-μl reservoir solution consisting of 0.4 M trisodium citrate, 4% (w/v) PEG3350, and 10% (w/v) xylitol, within 1 week. To improve the diffraction quality of crystals, TcpB-TcpF crystals were dehydrated by transferring to a new well and equilibrating to a fresh reservoir solution consisting of 0.15 M trisodium citrate, 20% (w/v) PEG3350, and 10% (w/v) xylitol for 3 days. X-ray diffraction data were collected on beamline BL38B1 at SPring-8 (Hyogo, Japan). All crystals were flash-frozen in a nitrogen gas stream at 100 K. All collected diffraction data were indexed and processed using XDS (*46*) and scaled using Aimless from the CCP4 program suite (*47*). Data collection and processing statistics are presented in table S1.

The initial TcpB phases were calculated using the single anomalous dispersion method involving the SeMet derivative crystal via Autosol in the PHENIX software package (*48*–*50*). Model building and refinement procedures were performed using the programs Coot (*51*) and phenix.refine from PHENIX. The resulting model was used as the search model for MR via the program PHASER in the PHENIX software package using x-ray data from the crystal of wild-type TcpB (*52*). The TcpB model was further refined using the program phenix.refine, resulting in a final model with *R*_work_ and *R*_free_ values of 0.174 and 0.208, respectively. The final TcpB model geometry was verified using MolProbity (*53*). Phase determination and refinement statistics are listed in table S1.

The initial TcpB-TcpF (1–15) phases were calculated by MR using PHASER in PHENIX. The TcpB crystal structure was used as a search model. The MR solution contained two TcpB trimers. Following several refinement cycles using phenix.refine in PHENIX and Coot, interpretable electron density maps of the interface between two molecules of the TcpB trimer were produced. Bulky Phe^1^ and Tyr^5^ electron densities of the TcpF (1–15) peptide were observed in the interface. The TcpF (1–15) was modeled from these Phe^1^ and Tyr^5^ residues as a starting point using Coot. We finally modeled the 11 N-terminal residues of TcpF, which consisted of Phe^1^ to Val^11^, at this density. Moreover, the TcpB-TcpF (1–15) model was further refined using phenix.refine and Coot, resulting in a final model with *R*_work_ and *R*_free_ values of 0.243 and 0.292, respectively. The geometry of the TcpB-TcpF (1–15) model was verified using MolProbity. The phase determination and refinement statistics are listed in table S1.

The initial phases of TcpB-TcpF were calculated by MR using PHASER in PHENIX. The crystal structures of TcpB-TcpF (1–15) and TcpF (code: 3oc5) were used as search models. The MR solution contained one TcpB-TcpF (1–15) complex and three TcpF molecules. Several refinement cycles produced an unmodeled density in one monomer of the TcpF trimer. On the basis of this improved electron density, we modeled an additional loop structure using Tyr^12^ to Glu^25^ of TcpF between the NTD of TcpF and TcpF (1–15) fragments. Eventually, one TcpF molecule closest to the TcpB trimer was constructed as a full-length model, whereas the other two TcpF molecules were not completely constructed. The TcpB-TcpF model was further refined using phenix.refine and Coot, resulting in a final model with *R*_work_ and *R*_free_ values of 0.292 and 0.314, respectively. The model geometry was evaluated using MolProbity software. Phase determination and refinement statistics are listed in table S1.

### Sequence and phylogenetic analyses

To evaluate the evolutionary relationships between TcpF and other (predicted) secreted proteins, we investigated the presence of secreted T4Pb proteins in other bacteria. We have previously reported some minor T4bP pilins by BLASTP analysis using domain 3 of the CofB protein sequence (*30*). The protein sequences of the predicted secreted proteins were obtained from registered genome sequences by referring to the operon structure of T4bP, *tcp* operon, or *cof* operon. Selected protein sequences from different bacteria were used for multiple protein sequence alignments with TcpF. A multiple alignment was generated using the MEGA X (*54*) implementation of MUSCLE. The evolutionary history was inferred using the neighbor-joining method. The optimal tree with the sum of branch length = 7.31350002 is shown. The tree is drawn to scale, with branch lengths in the same units as those of the evolutionary distances used to infer the phylogenetic tree. The evolutionary distances were computed using the Poisson correction method and are in units of a number of amino acid substitutions per site. The analysis involved 15 protein sequences. All positions containing gaps and missing data were eliminated. There was a total of 419 positions in the final dataset. Evolutionary analyses were conducted in MEGA X (*54*).

### Molecular modeling of TCP alone and in complex with TcpF

The TcpA filament model containing 18 subunits was initially built using the ETEC CFA/III model (*24*, *30*, *34*, *55*), previously generated using cryo–electron microscopy data from the *V. cholerae* TCP (*32*). A full-length model of TcpA containing a hydrophobic N-terminal helix was constructed by homology modeling using the CofA full-length model and MODELER 9.24 (*57*). The modeled TcpA molecules were superimposed on and replaced by the 18 CofA subunits in the CFA/III model. We confirmed that the constructed TcpA filament model fits the electron density map of TCP (*32*) using Chimera (*58*). The TCP model, in which the trimeric structure of the minor pilin TcpB is on top of the TcpA filament model, was generated on the basis of homology modeling, computational docking, and molecular dynamics (MD) calculations. The hydrophobic N-terminal helix of TcpB domain 1 was achieved by homology modeling using the CofB domain 1 α1 model. Three models of TcpB domain 1 were superimposed on and replaced by the three TcpA molecules located on the top of the TcpA filament model. We next added the remaining structure, domain 2 and domain 3 of the TcpB trimer, above the model and connected the linker between domain 1 and domain 2 using Coot (*51*). The energy-minimization process was further performed until the energy of the system converged to 100 kJ mol^−1^ nm^−1^ with the quasi-Newton method in the GROMACS 2020 program package (*59*). The energy calculation was performed with the AMBER99SB-ILDN energy terms under the NPT ensemble and periodic boundary conditions (*60*). Simple point-charge water molecules were chosen for the water model with six Na^+^ counterions added to attain an electrically neutral system. In this resulting model, several critical interactions previously predicted to involve TCP formation are observed between the positively charged N-terminal amine of Met^1^ in α1 of TcpA and Glu^5^ in α1 of TcpA, and between Arg^26^ in α1 of TcpA and Glu^83^ in α/β-loop of TcpA. We also confirmed the interactions between the amine of Met^1^ in α1 of TcpB and Glu^5^ in α1 of TcpA, and between Arg^26^ in α1 of TcpB and Glu^83^ in the α/β-loop of TcpA, which correspond to the interactions in the TcpA filament. The TcpF-TCP filament model was constructed by superimposing domain 2 and domain 3 of the TcpB trimer in the TcpB-TcpF complex on the corresponding part of the TcpB trimer located at the top of the TCP filament model. The N-terminal missing part (Ala^16^ to Gly^23^ in TcpF[B] or Ala^13^ to Glu^25^ in TcpF[C]) of two of the three TcpF molecules was also modeled by MODELLER 9.24 (*57*). The resulting model of the TcpF-TCP complex was further refined by energy minimization without structural constraints. The energy-minimization process was performed until the energy of the system converged to 100 kJ mol^−1^ nm^−1^ with the quasi-Newton method in GROMACS 2020 (*59*). The energy calculation was performed with the AMBER99SB-ILDN energy terms under the NPT ensemble and periodic boundary conditions. Simple point-charge water molecules were chosen for the water model with 24 Na^+^ counterions added to attain an electrically neutral system.

## Notes

View/request a protocol for this paper from *Bio-protocol*.
