# Supplementary material for: Structural basis for the toxin-coregulated pilus–dependent secretion of Vibrio cholerae colonization factor
Source: Sci Adv. 2022 Oct 14;8(41):eabo3013. doi: 10.1126/sciadv.abo3013 (PMC9565799; doi:10.1126/sciadv.abo3013)
Supplement: Supplementary file 1 — Figs. S1 to S14 Tables S1 and S2 [file sciadv.abo3013_sm.pdf]

Science Advances
AAAS

Supplementary Materials for

Structural basis for the toxin-coregulated pilus–dependent secretion of *Vibrio cholerae* colonization factor

Hiroya Oki *et al.*

Corresponding author: Shota Nakamura, nshota@gen-info.osaka-u.ac.jp

Sci. Adv. **8**, eabo3013 (2022)
DOI: 10.1126/sciadv.abo3013

This PDF file includes:

Figs. S1 to S14
Tables S1 and S2

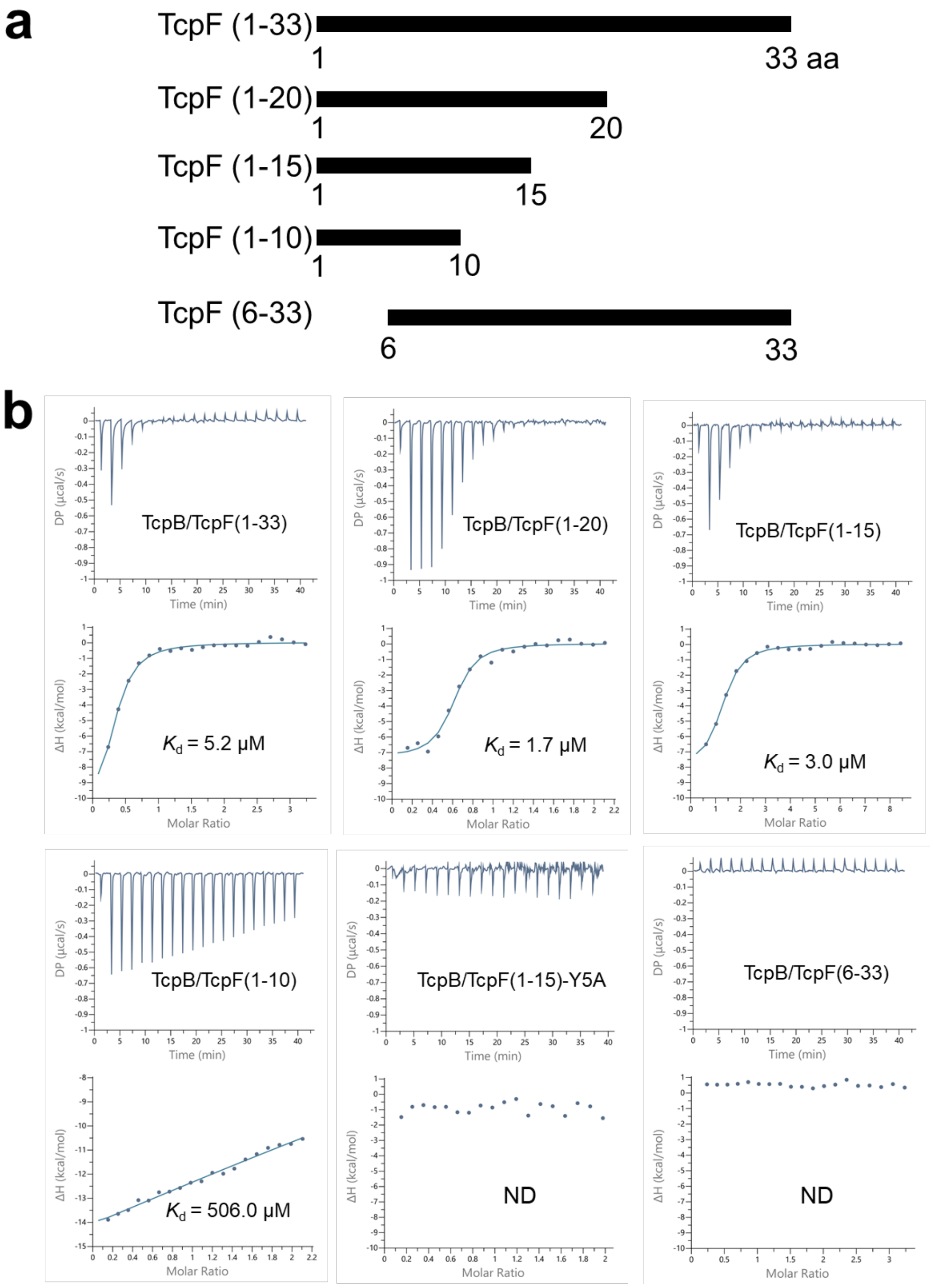

Fig. S1. Interaction analysis between TcpB and the N-terminal fragments of TcpF. (a) Diagram showing the peptides of the TcpF N-terminal region: TcpF(1-33), TcpF(1-20), TcpF(1-15), TcpF(1-10), and TcpF(6-33). (b) The isothermal titration calorimetry (ITC) profiles of TcpB titration with the peptides from the N-terminal of TcpF, as shown in Fig S1a, and the TcpF(1-15)-Y5A variant peptide. Each lower panel depicts an integrated heat plot for the titration.

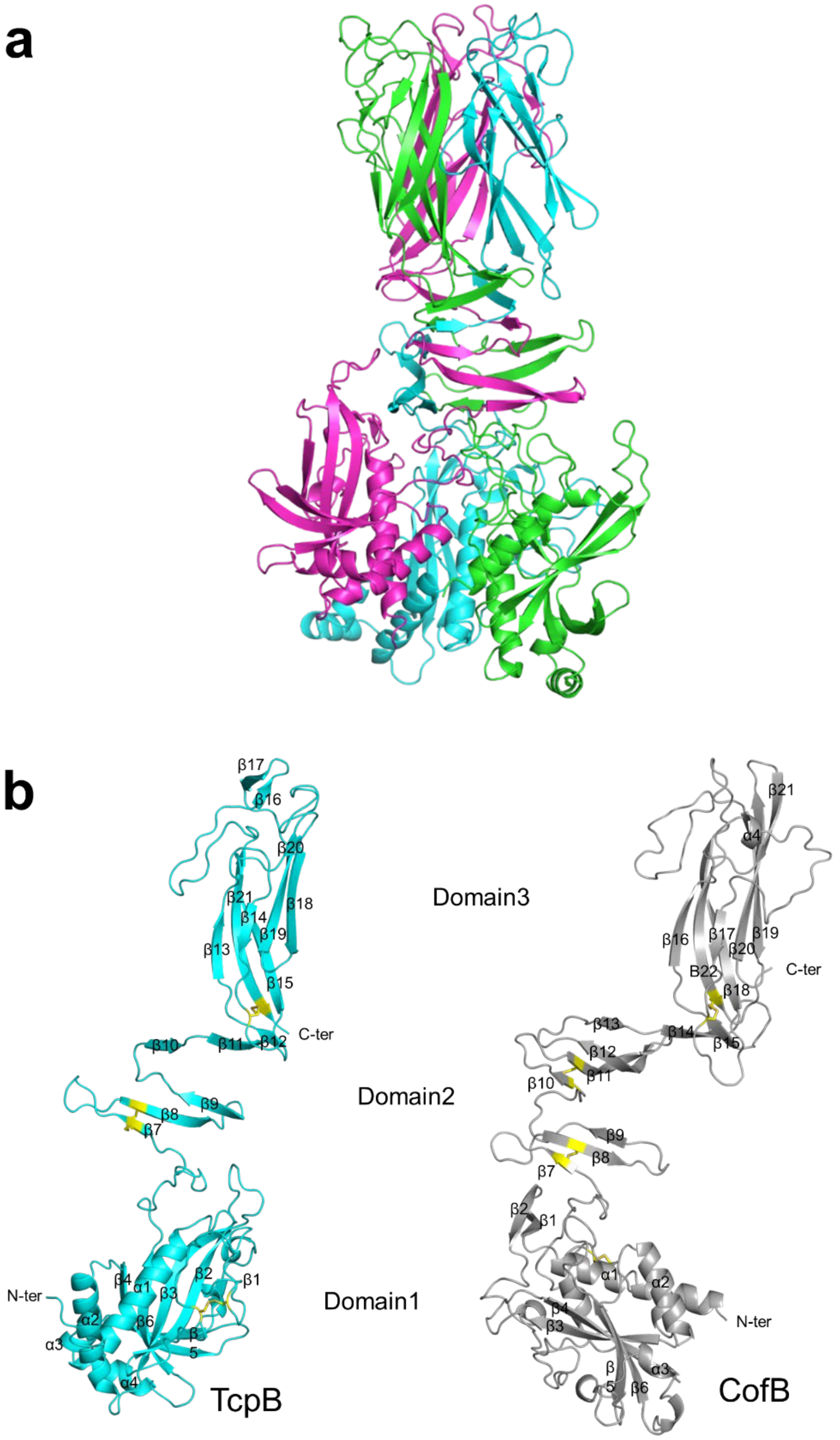

Fig. S2. Crystal structure of TcpB. (a) Ribbon model of the crystal structure of the apo TcpB homotrimer observed in the asymmetric unit. (b) Structural comparison between a TcpB monomer (left panel) and CofB monomer (right panel, pdb code: 5ax6). Each disulfide bond bridge is shown as a yellow stick model.

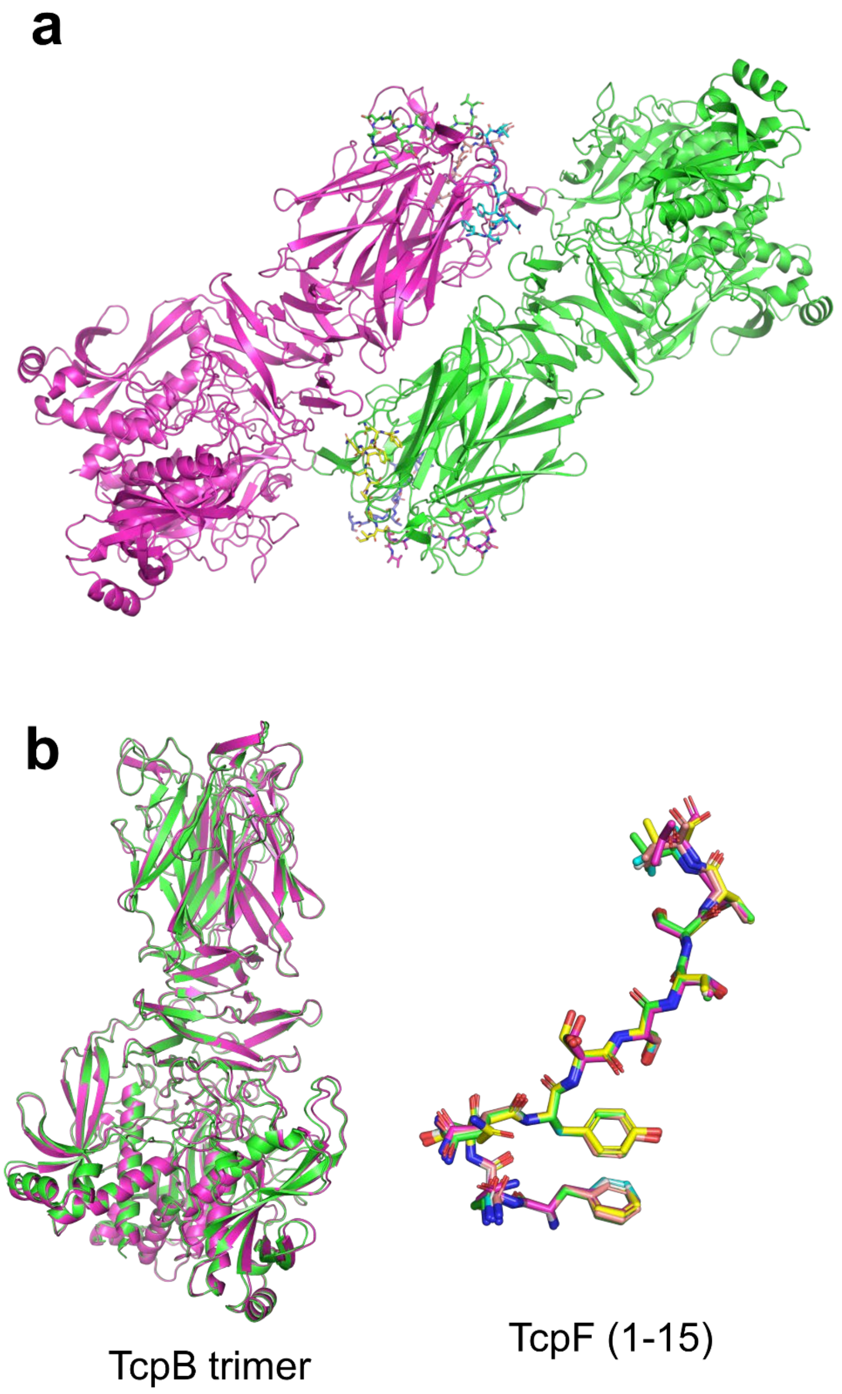

Fig. S3. Crystal structure of the TcpB-TcpF (1-15) complex. (a) Two TcpB-TcpF (1-15) complexes in the asymmetric unit. (b) Superposition of two TcpB homotrimer molecules (left panel) and six TcpF (1-15) peptides (right panel).

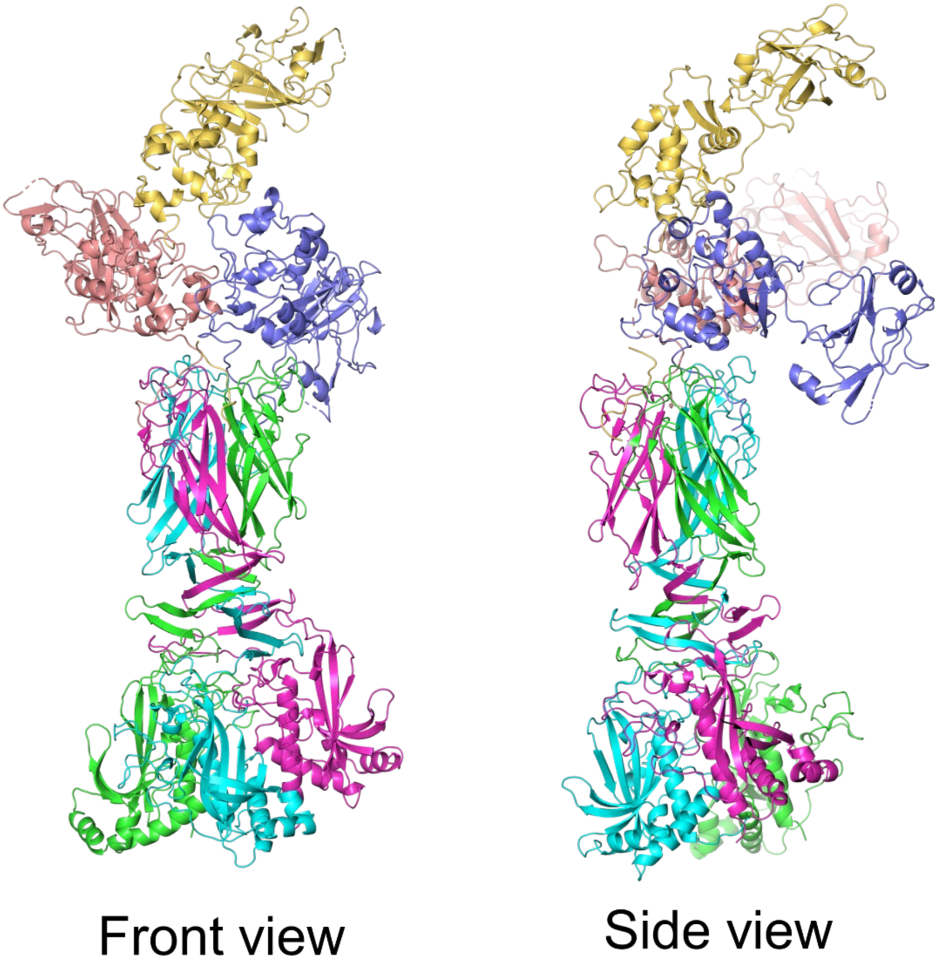

Fig. S4. Crystal structure of TcpB-TcpF. Front view (left panel) and side view (right panel) of the structure with the TcpB trimer (cyan, magenta, and green) and the bound TcpF trimer (blue, yellow, salmon pink) shown as ribbon models.

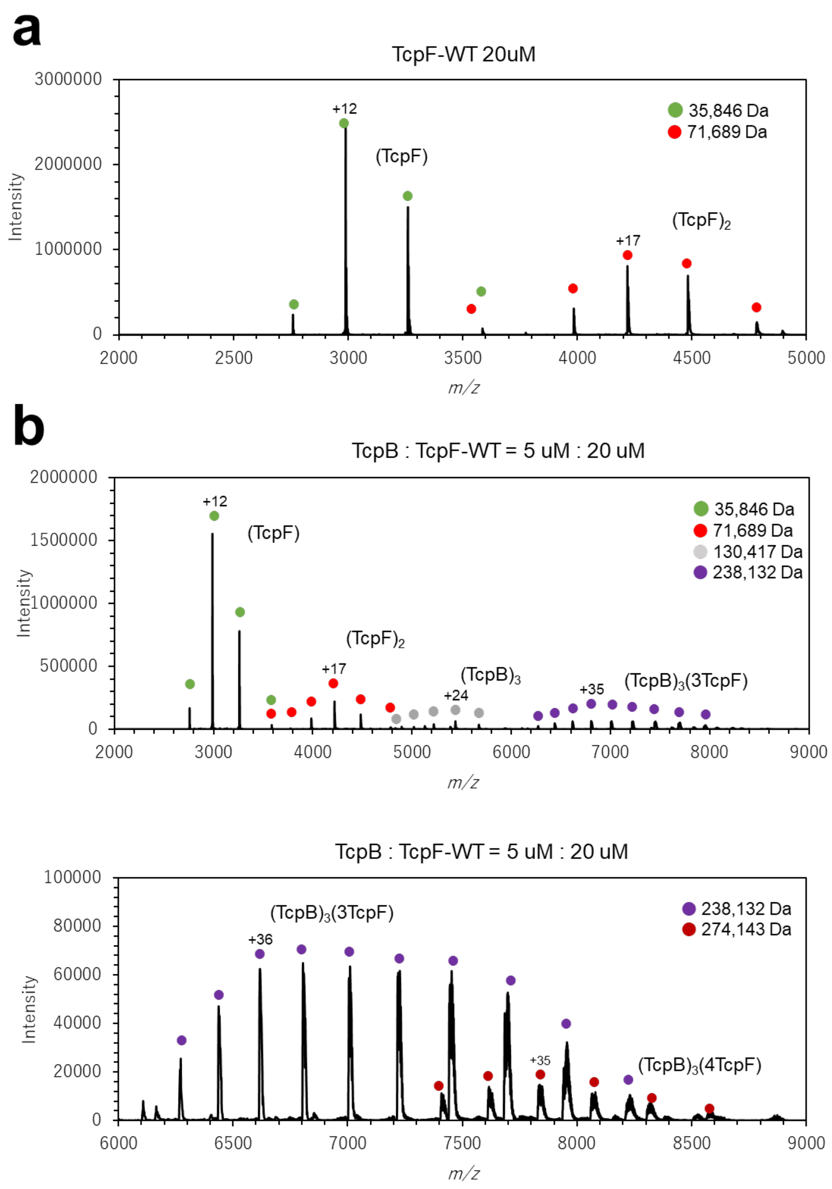

Fig. S5. Native mass spectrometry of TcpB-TcpF complexes. (a) Native mass spectrum of wild-type TcpF. Light green and light red circles show the ion series of the unbound TcpF monomer (TcpF) and TcpF dimer $(TcpF)_2$, respectively. (b) Native mass spectrum of a mixture of TcpB and wild-type TcpF at a 1:4 molar ratio (upper panel) with a zoom-in view (lower panel). The gray circle, purple circle, and red circle show the ion series of the TcpB trimer $(TcpB)_3$, TcpB trimer/three TcpF complexes $(TcpB)_3$(3TcpF), and TcpB trimer/four TcpF complexes $(TcpB)_3$(4TcpF), respectively.

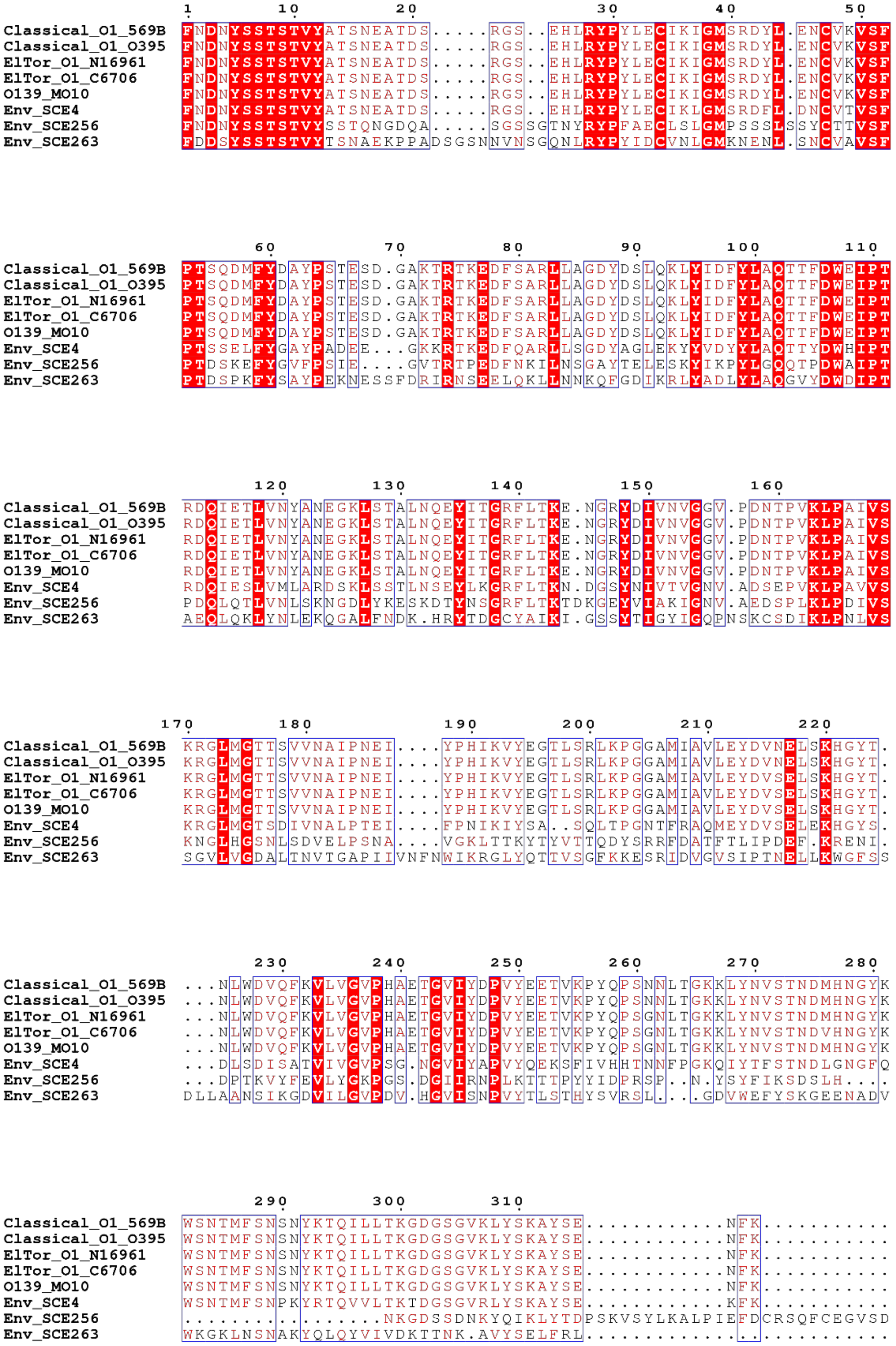

Fig. S6. Multiple alignments of the mature TcpF protein sequences among pathogenic and environmental *V. cholerae* strains. Strains used for the alignment were 569B (O1, classical,

Inaba, accession number: AAA27566.1), O395 (O1, classical, Ogawa, accession number: WP_001269569.1), N16961 (O1, El Tor, accession number: AAF94000.1), C6706 (O1, El Tor, accession number: QJS92980.1), MO10 (O139, accession number: QNE70259.1), SCE4 (environmental strain, accession number: AAG31789.1), SCE256 (environmental strain, accession number: AAG31794.1), and SCE263 (environmental strain, accession number: AAK57701.1). Numbers above the alignment are the amino acid numbers of the 569B strain. The figure was generated with ESPript (https://espript.ibcp.fr/ESPript/cgi-bin/ESPript.cgi).

a

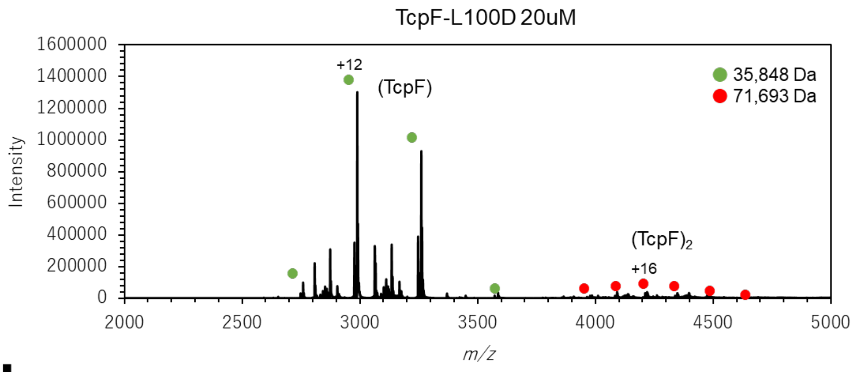

TcpF-L100D 20uM
35,848 Da
71,693 Da
+12
(TcpF)
(TcpF)2
+16
Intensity
m/z

b

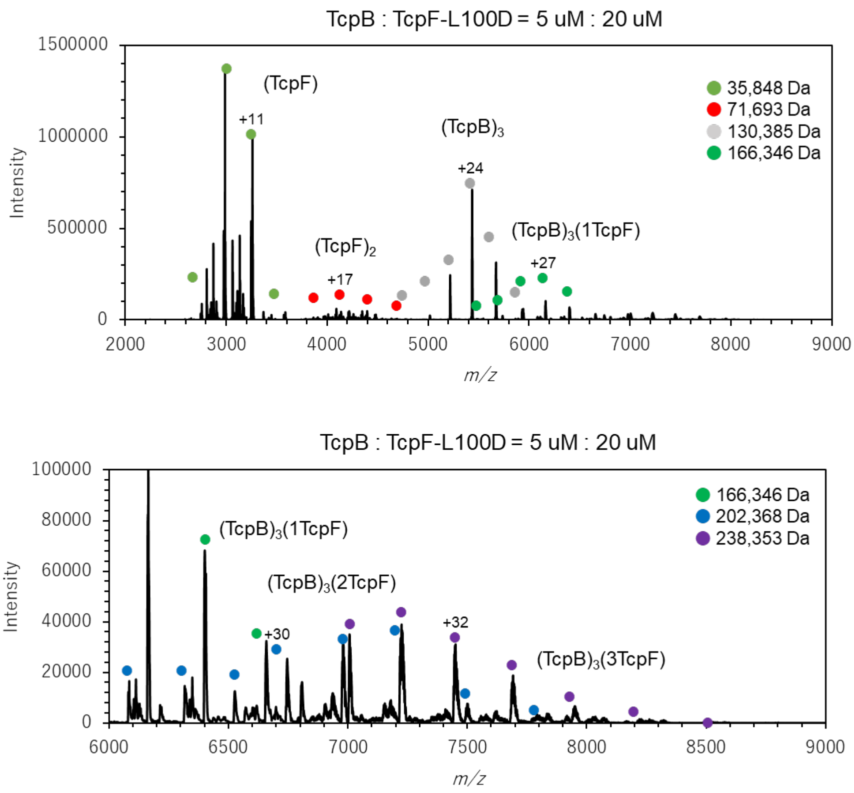

TcpB : TcpF-L100D = 5 uM : 20 uM
35,848 Da
71,693 Da
130,385 Da
166,346 Da
(TcpF)
+11
(TcpB)3
+24
(TcpB)3(1TcpF)
+27
(TcpF)2
+17
Intensity
m/z
TcpB : TcpF-L100D = 5 uM : 20 uM
166,346 Da
202,368 Da
238,353 Da
(TcpB)3(1TcpF)
(TcpB)3(2TcpF)
+30
+32
(TcpB)3(3TcpF)
Intensity
m/z

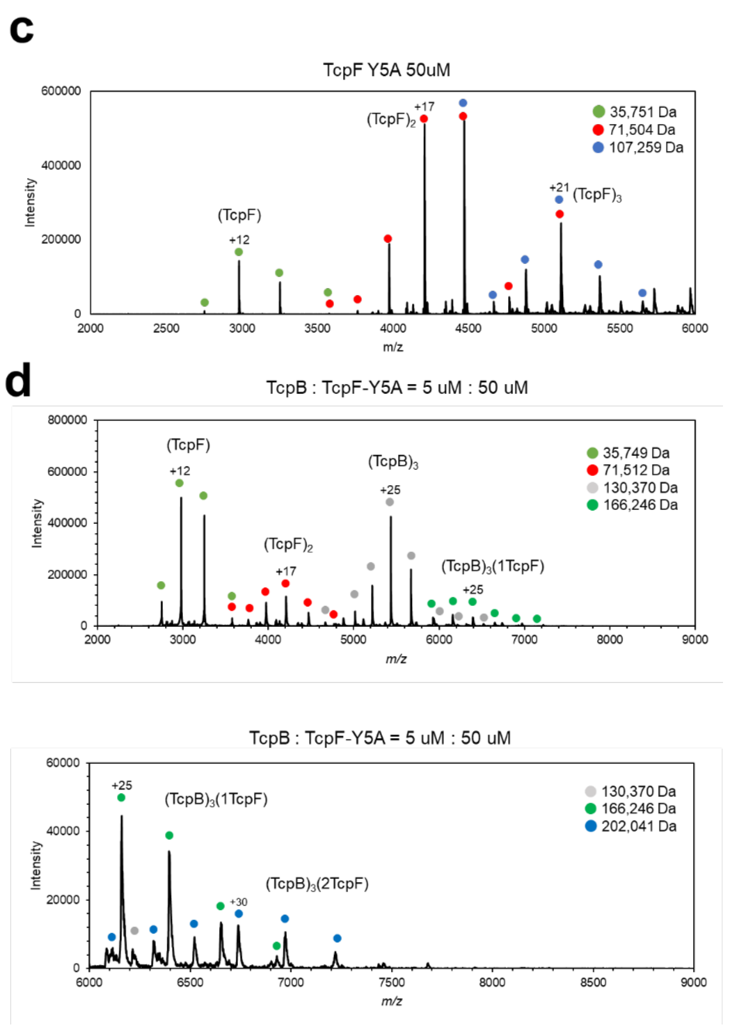

Fig. S7. Native mass spectrometry of TcpB-TcpF-L100D and TcpB-TcpF-Y5A. (a) Native mass spectrum of the TcpF-L100D mutant. (b) Native mass spectrum of a mixture of TcpB and the TcpF-L100D mutant at a 1:4 molar ratio (top panel) and the zoomed-in view (bottom panel). (c) Native mass spectrum of the TcpF-Y5A mutant. (d) Native mass spectrum of a mixture of TcpB and the TcpF-Y5A mutant at a 1:10 molar ratio (top panel) and zoomed-in view (bottom panel). The light green circle, light red circle, and light blue circle show the ion series of the unbound TcpF monomer (TcpF), TcpF dimer $(TcpF)_2$, and TcpF trimer $(TcpF)_3$, respectively. The gray circle, pink circle, green circle, blue circle, and purple circle show the ion series of the TcpB trimer $(TcpB)_3$, TcpB hexamer $(TcpB)_6$, TcpB trimer/one TcpF complex $(TcpB)_3$(1TcpF), TcpB trimer/two TcpF complexes $(TcpB)_3$(2TcpF), and TcpB trimer/three TcpF complexes $(TcpB)_3$(3TcpF), respectively.

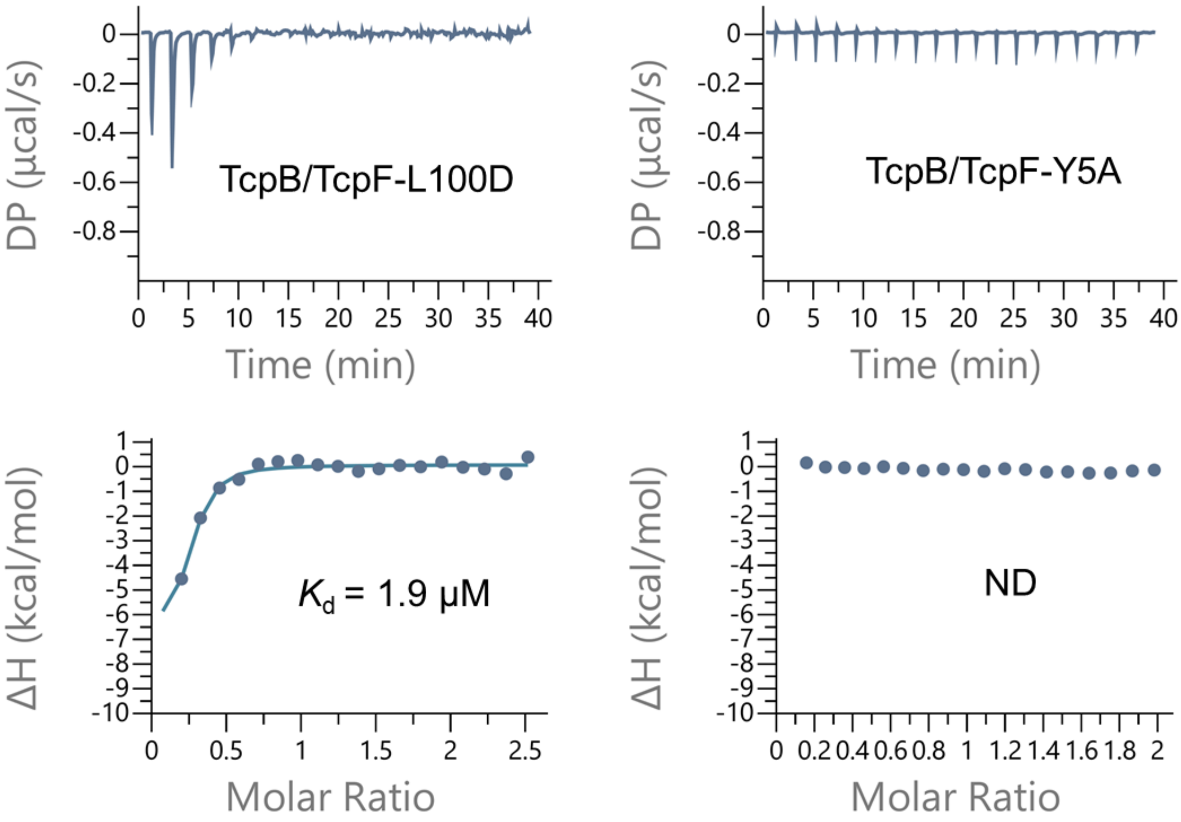

Fig. S8. Interaction analysis between TcpF-L100D or the TcpF-Y5A mutant and TcpB. Left top panel: isothermal titration calorimetry (ITC) profiles of TcpB titration with TcpF-L100D. Right top panel: ITC profiles of TcpB titration with TcpF-Y5A. Each lower panel depicts an integrated heat plot of the titration.

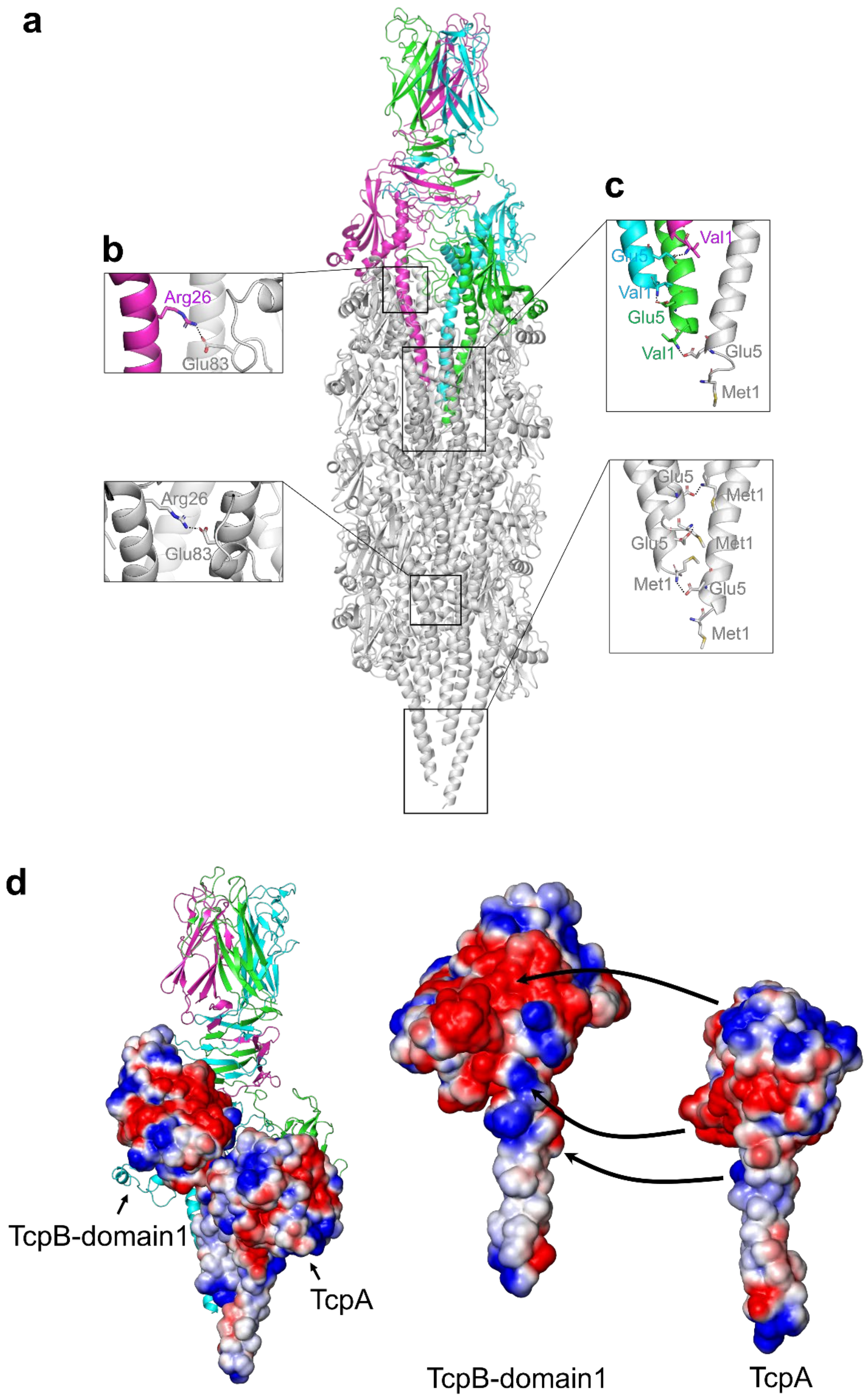

Fig. S9. TCP filament model. (a) Side view of the toxin-coregulated pilus (TCP) model depicted as a ribbon model. (b) Close-up views of the stabilizing salt bridges between Arg26

(TcpB) and Glu83 (TcpA), and between Arg26 (TcpA) and Glu83 (TcpA). (c) Close-up views of the electrostatic interactions between the amine of Val1 (TcpB) and Glu5 (TcpA) and between the amine of Met1 (TcpA) and Glu5 (TcpA). (d) Electrostatic surface potential representations of the interaction interface between one TcpB-domain 1 and the interacting TcpA, as calculated using the APBS tool in PyMOL (http://www.pymol.org/pymol). In addition to the interaction between the positively charged amine of the N-terminal and Glu5 of two α1s, the TcpB-TcpA interaction is presumably promoted by the shape and charge complementarity of globular domains, such as the electrostatic interaction between Arg26 in α1 of TcpB and Glu83 in α/β-loop of TcpA, corresponding to the Arg26-Glu83 pairwise interaction previously shown as critical in the TcpA filament.

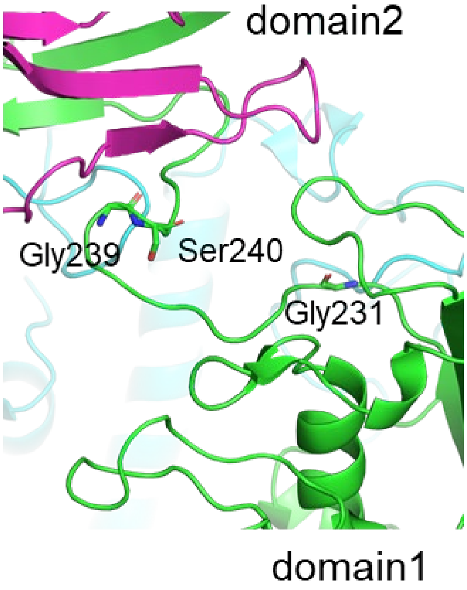

Fig. S10. Zoomed-in view of the linker between TcpB domain 1 and domain 2 (green). In this case, the two Gly residues (Gly231 and Gly239) and one Ser residue (Ser240) are depicted as a stick model.

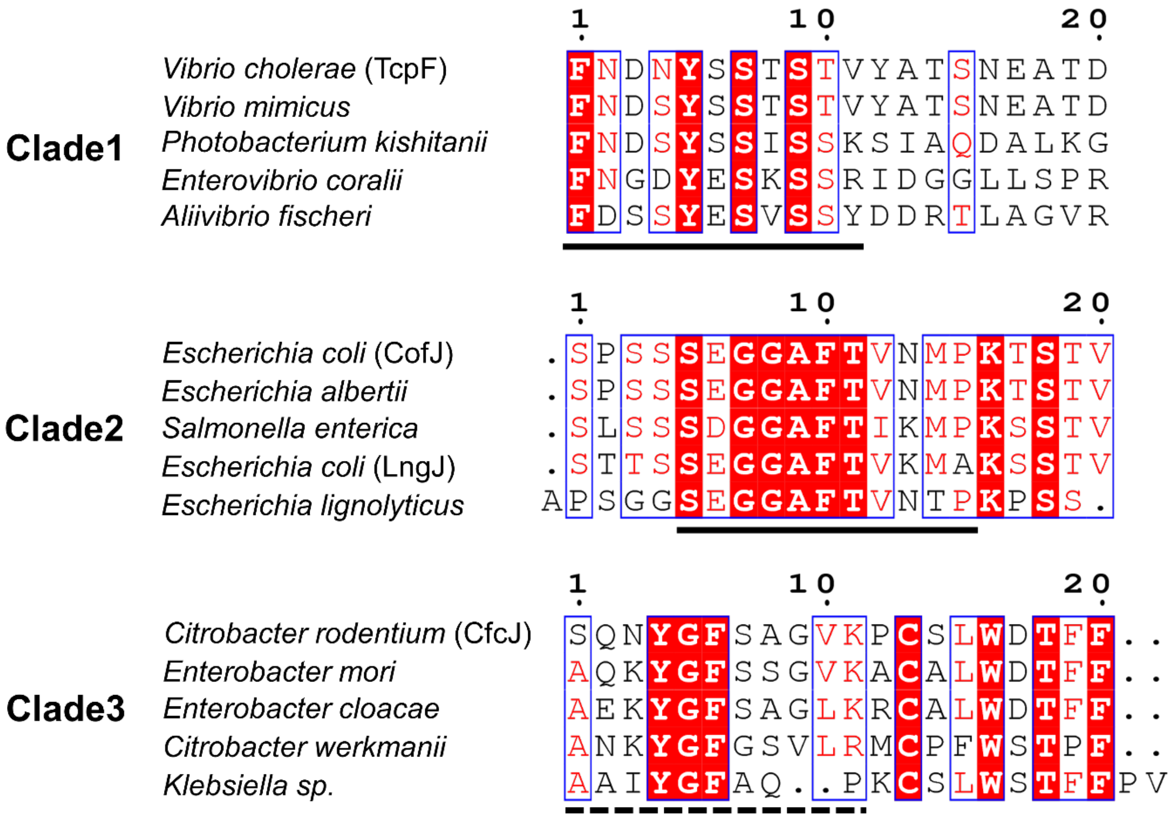

Fig. S11. Protein sequence alignments among secreted proteins in T4bP. A sequence alignment of the 20 N-terminal amino acids of *Vibrio cholerae* TcpF and putative secreted proteins from *Vibrio mimicus*, *Photobacterium kishitanii*, *Enterovibrio coralii*, and *Allivibrio fischeri* in Clade 1. Middle panel: A sequence alignment of the 20 N-terminal amino acids of *Escherichia coli* CofJ, *Escherichia coli* LngJ, predicted secreted proteins from *Escherichia albertii*, *Escherichia lignolyticus*, and *Salmonella enterica* in Clade 2. Lower panel: A sequence alignment of the 20 N-terminal amino acids among *Citrobacter rodentium* CfcJ, predicted secreted proteins from *Enterobacter mori*, *Enterobacter cloacae*, *Klebsiella sp.*, and *Citrobacter werkmanii* in Clade 3. The details of the clades and accession number of amino acid sequences are shown in Fig. 4d. The mature N-terminal sequences of secreted proteins generated from N-terminal signal sequence cleavage were predicted by SignalP-6.0 (https://services.healthtech.dtu.dk/service.php?SignalP). The black underlines indicate the position of T4bP-SS of TcpF or CofJ. The black dashed underline indicates the predicted position of T4bP-SS in CfcJ. The figures were generated with ESPript (https://espript.ibcp.fr/ESPript/cgi-bin/ESPript.cgi).

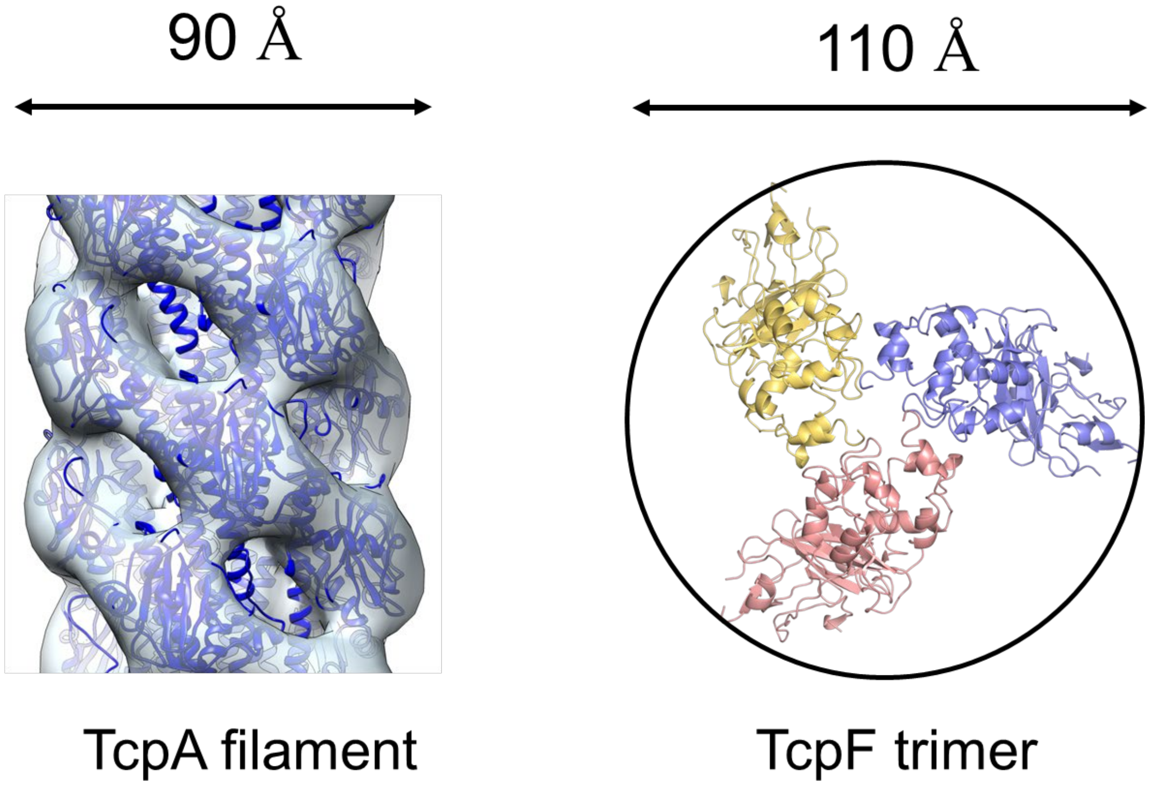

Fig. S12. Comparison of the diameter of the toxin-coregulated pilus (TCP) model and TcpF trimer. Left panel: Superposition of a TcpA filament model shown as a blue ribbon model with the electron density map of TCP (ID: EMD-1955). The diameter of this filament model is approximately 90 Å. Right panel: model of the top view of the TcpF trimer. The maximum diameter of the TcpF trimer is approximately 110 Å.

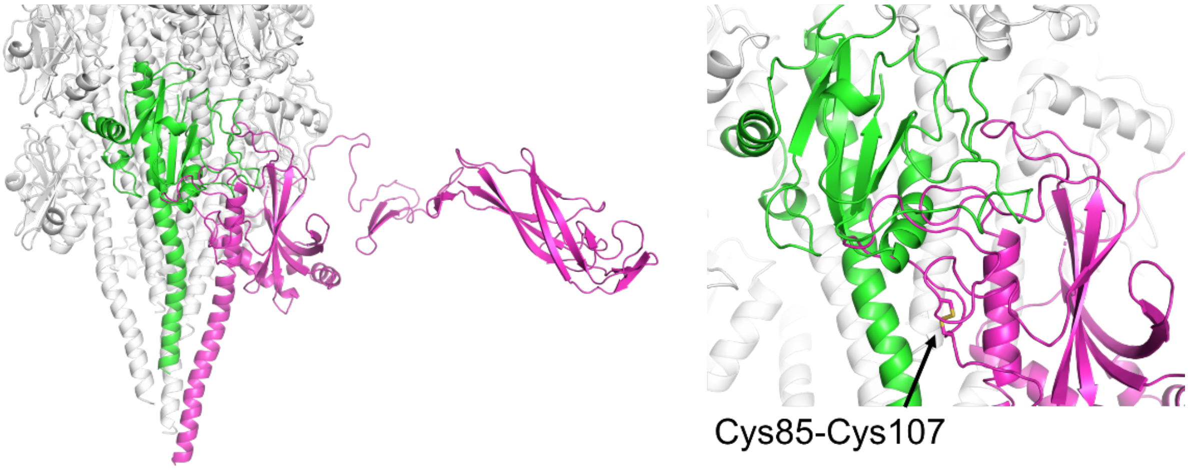

Fig. S13. Structural model of the TcpA filament–TcpB monomer complex. Left panel: Side view of the TcpA filament–TcpB monomer complex depicted as a ribbon model, constructed by superimposing the TcpB monomer model (magenta) onto a TcpA molecule (green) at the bottom of the TcpA filament model (gray) with Coot (*51*) and PyMOL. The α/β loop of TcpB, which has a disulfide bond (Cys85-Cys107) and is longer than that of TcpA, collides with the D-region of TcpA (right panel).

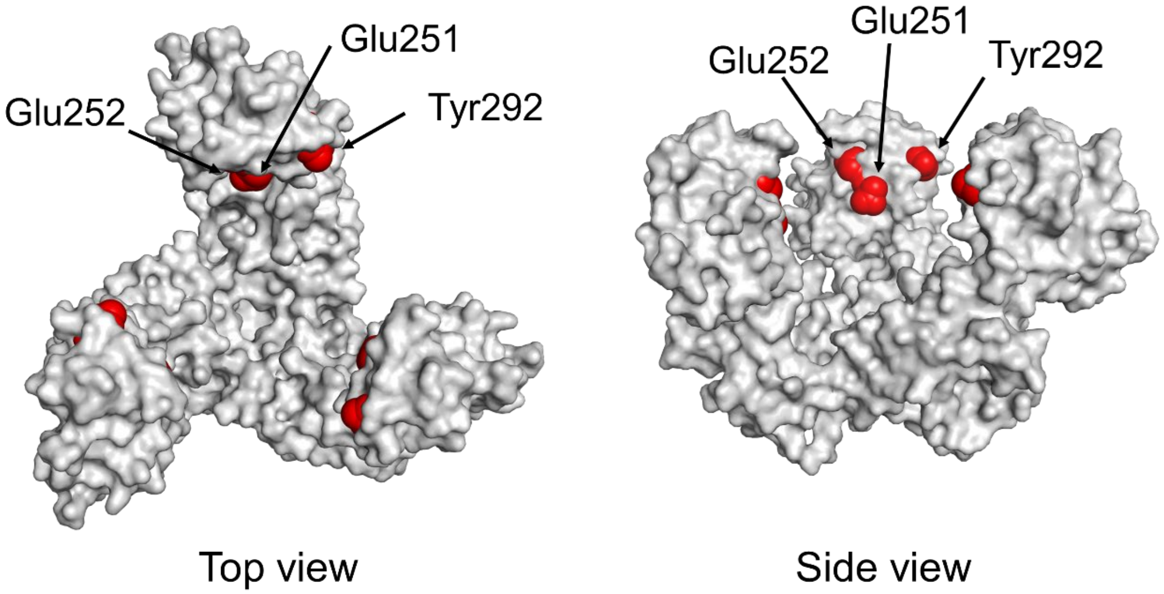

Fig. S14. Location of residues important for *V. cholerae* colonization. Top view (left) and side view (right) of the TcpF trimer with the surface model. Glu251, Glu252, and Tyr292 of TcpF, which are important for *V. cholerae* colonization, are depicted in red.

Table S1. Crystallographic data collection and refinement statistics

Data collection	TcpB		TcpB-TcpF(1-15)	TcpB-TcpF
	Native	SeMet derivative		
Beamline	SPring-8 BL38B1	SPring-8 BL38B1	SPring-8 BL38B1	SPring-8 BL38B1
Detector	Pilatus3 6M	Pilatus3 6M	Pilatus3 6M	Pilatus3 6M
Wavelength (Å)	1.00000	0.97854	1.00000	1.00000
Space group	*I 4 2 2*	*I 4 2 2*	$P2_12_12_1$	$P\,6_322$
Unit-cell parameters (Å, º)	$a = b = 170.83$, $c = 262.86$, $\alpha = \beta = \gamma = 90.00$	$a = b = 170.54$, $c = 262.41$, $\alpha = \beta = \gamma = 90.00$	$a = 74.88$, $b = 128.55$, $c = 327.24$, $\alpha = \beta = \gamma = 90.00$	$a = b = 281.14$, $c = 297.60$, $\alpha = \beta = 90.00$, $\gamma = 120.00$
Total measured reflections	3267250 (183488)	1871727 (121942)	1413980 (71962)	1100587 (87635)
Unique reflections	83755 (4522)	70934 (4514)	139539 (6930)	57983 (4439)
Resolution (Å)	47.75-2.32 (2.36-2.32)	47.66-2.45 (2.51-2.45)	48.77-2.30 (2.34-2.30)	49.21-4.05 (4.16-4.05)
R_{merge}	0.207 (2.271)	0.173 (1.791)	0.084 (1.069)	0.170 (3.072)
$CC_{1/2}$	0.999 (0.856)	0.999 (0.848)	0.997 (0.851)	0.999 (0.533)
Completeness (%)	100.0 (100.0)	100.0 (100.0)	99.1 (99.7)	99.9 (100.0)
Wilson *B*-factor ($Å^2$)	47.9	42.3	52.6	185.5
Average $I/\sigma(I)$	15.3 (1.9)	18.4 (2.3)	12.1 (2.0)	13.1 (1.3)
Redundancy	39.0 (40.6)	26.4 (27.0)	10.1 (10.4)	19.0 (19.7)
**Refinement**				
Resolution (Å)	23.37-2.32 (2.35-2.32)		37.20-2.30 (2.33-2.30)	31.83-4.05 (4.10-4.05)
Reflections	83639 (2758)		139216 (4649)	139216 (1884)

No. Atoms			
Protein	9114	18768	16441
Water	717	362	-
Other	170	33	-
R_{work}	0.174 (0.245)	0.222 (0.329)	0.274 (0.407)
R_{free}	0.208 (0.317)	0.264 (0.343)	0.301 (0.395)
R.M.S.D. from ideal			
Bonds (Å)	0.009	0.009	0.004
Angles (º)	1.183	1.072	0.737
B-factors (Å^2)	53.0	66.0	226.0
Ramachandran plot analysis (%)			
Most favored	98.2	96.4	94.9
Allowed	1.8	3.3	4.7
PDB code	7W63	7W64	7W65

Highest-resolution shell statistics are in parentheses.

Table S2. Sequences of primers used in the study

Primer	Primer sequence (5′-3′)
tcpA_Forward	CGACAGCCAGAATATGACCAAAGCC
tcpA_Reverse	CTCGAGTTAGCTATTGCCAAATGCCACG
tcpB_ Forward	CGAACTGATGATCAAATCTTCTAATGCG
tcpB_ Reverse	ATGCTCGAGTTAGTTCTCACACCACTGGAACG
tcpF_ Forward	CTTCAACGATAACTATAGCAGCACCAG
tcpF_ Reverse	CTCGAGTTATTTAAAATTTTCGCTATAGGCCTTGC
tcpFF_Forward	TTCAACGATAACTATAGCAGCACCAGC
tcpFF_Reverse	CCTACCTTCGATGCCACCCTGGAAGTACAGGTTTTC
tcpF_Y5A_Forward	GTTCAACGATAACGCCAGCAGCACCAGC
tcpF_Y5A_Reverse	GCTGGTGCTGCTGGCGTTATCGTTGAAC
tcpF_L100D_Forward	CTGTATATTGATTTCTATGACGCACAGACCACCTTT GAC
tcpF_L100D_Reverse	GTCAAAGGTGGTCTGTGCGTCATAGAAATCAATAT ACAG
